# Why physiology will continue to guide the choice between balanced crystalloids and normal saline: a systematic review and meta-analysis

**DOI:** 10.1186/s13054-019-2658-4

**Published:** 2019-11-21

**Authors:** Charlotte L. Zwager, Pieter Roel Tuinman, Harm-Jan de Grooth, Jos Kooter, Hans Ket, Lucas M. Fleuren, Paul W. G. Elbers

**Affiliations:** 1 Department of Intensive Care Medicine, Amsterdam UMC, Location VUmc, Vrije Universiteit Amsterdam, Research VUmc Intensive Care (REVIVE), Amsterdam Medical Data Science (AMDS), Amsterdam Cardiovascular Science (ACS), Amsterdam Infection and Immunity Institute (AI&II), De Boelelaan 1117, 1081 HV Amsterdam, The Netherlands; 2 Department of Internal Medicine, Amsterdam UMC, Location VUmc, Vrije Universiteit Amsterdam, Research VUmc Intensive Care (REVIVE), Amsterdam Medical Data Science (AMDS), Amsterdam Cardiovascular Science (ACS), Amsterdam Infection and Immunity Institute (AI&II), De Boelelaan 1117, 1081 HV Amsterdam, The Netherlands; 3 University Library, Amsterdam UMC, Location VUmc, Vrije Universiteit Amsterdam, Research VUmc Intensive Care (REVIVE), Amsterdam Medical Data Science (AMDS), Amsterdam Cardiovascular Science (ACS), Amsterdam Infection and Immunity Institute (AI&II), De Boelelaan 1117, 1081 HV Amsterdam, The Netherlands

**Keywords:** Balanced crystalloids, Normal saline, Intravenous fluid administration, Intensive care unit, Emergency department, Meta-analysis, Trial sequential analysis, Required information size, Physiology

## Abstract

**Background:**

Crystalloids are the most frequently prescribed drugs in intensive care medicine and emergency medicine. Thus, even small differences in outcome may have major implications, and therefore, the choice between balanced crystalloids versus normal saline continues to be debated. We examined to what extent the currently accrued information size from completed and ongoing trials on the subject allow intensivists and emergency physicians to choose the right fluid for their patients.

**Methods:**

Systematic review and meta-analysis with random effects inverse variance model. Published randomized controlled trials enrolling adult patients to compare balanced crystalloids versus normal saline in the setting of intensive care medicine or emergency medicine were included. The main outcome was mortality at the longest follow-up, and secondary outcomes were moderate to severe acute kidney injury (AKI) and initiation of renal replacement therapy (RRT). Trial sequential analyses (TSA) were performed, and risk of bias and overall quality of evidence were assessed. Additionally, previously published meta-analyses, trial sequential analyses and ongoing large trials were analysed for included studies, required information size calculations and the assumptions underlying those calculations.

**Results:**

Nine studies (*n* = 32,777) were included. Of those, eight had data available on mortality, seven on AKI and six on RRT. Meta-analysis showed no significant differences between balanced crystalloids versus normal saline for mortality (*P* = 0.33), the incidence of moderate to severe AKI (*P* = 0.37) or initiation of RRT (*P* = 0.29). Quality of evidence was low to very low. Analysis of previous meta-analyses and ongoing trials showed large differences in calculated required versus accrued information sizes and assumptions underlying those. TSA revealed the need for extremely large trials based on our realistic and clinically relevant assumptions on relative risk reduction and baseline mortality.

**Conclusions:**

Our meta-analysis could not find significant differences between balanced crystalloids and normal saline on mortality at the longest follow-up, moderate to severe AKI or new RRT. Currently accrued information size is smaller, and the required information size is larger than previously anticipated. Therefore, completed and ongoing trials on the topic may fail to provide adequate guidance for choosing the right crystalloid. Thus, physiology will continue to play an important role for individualizing this choice.

## Background

Intravenous fluids are the most frequently prescribed drugs in intensive care medicine and emergency medicine [1, 2]. Therefore, even small differences in outcomes related to the choice of fluids will have major clinical impact worldwide. This has fuelled the debate on the ideal composition of these fluids. In particular, the traditional choice of normal saline over balanced crystalloids has come into question.

Compared to normal saline, also known as 0.9% sodium chloride or saline, balanced crystalloids are more similar to human plasma, contain less chloride and have a higher in vivo strong ion difference [3]. There have been accumulating signals of harm associated with the use of normal saline over balanced crystalloids from physiological, preclinical and retrospective studies. These include findings of increased acidemia, reduced renal and gastric blood flow, reduced urine output, impaired renal function, increase inflammation, vasodilation, reduced response to inotropes and increased mortality [4].

However, subsequent randomized controlled clinical trials and meta-analyses failed to find clinically or statistically significant differences on relevant outcome measures [5–15]. This prompted the conduct of two very large trials, one in the setting of intensive care medicine [15] and the other in the setting of emergency medicine [16]. These trials did find a significant improvement in adjusted analyses, favouring balanced crystalloids in the composite outcome of Major Adverse Kidney Events within 30 days (MAKE30). However, no significant differences could be shown for its individual components: death, new renal replacement therapy or persistent renal dysfunction. Several attempts to also include these large trials into meta-analysis and trial sequential analysis have been made [14, 17, 18]. However, all of these exclusively focussed on the setting of intensive care medicine, made very different assumptions for calculating required information size and failed to adjust their analysis for the cluster design of included trials.

Therefore, we now set out to perform a rigorous systematic review, meta-analysis and trial sequential analysis focussing both on the setting of intensive care medicine and emergency medicine as these frequently represent a continuum of care for critically ill patients or those at risk to become critically ill. We also sought to compare our findings to those of other meta-analyses, trial sequential analyses and planned or ongoing large studies in the field.

Thus, the objective of our article was to examine the available evidence regarding the effect of balanced crystalloids versus normal saline on clinical outcomes (i.e. mortality at the longest follow-up, AKI and the need for RRT) in the setting of intensive care medicine and emergency medicine. Moreover, this report aims to provide a more precise estimate of the true current level of evidence and the potential contribution and relevance of future trials on choosing balanced crystalloids or normal saline in the setting of intensive care medicine and emergency medicine*.* This will allow us to address the ultimate question: can future trials be expected to provide definitive answers any time soon or will physiology continue to prevail when choosing between normal saline or balanced crystalloids?

## Methods

Our systematic review was performed according to our protocol registered at the international prospective register of systematic reviews (PROSPERO; no. CRD42018098845). The results are reported according to the preferred reporting items for systematic reviews and meta-analyses (PRISMA) guidelines [19]. The PRISMA checklist may be found in Additional file 1: Table S1.

### Eligibility criteria

Only randomized controlled trials (RCTs) that compared normal saline versus balanced crystalloids in the setting of intensive care medicine or emergency medicine were considered. Unpublished trials and trials published as abstracts were also considered for inclusion provided that adequate information on methods and results could be obtained. Normal saline was defined as 0.9% saline with a chloride content of 154 mmol/l and an in vivo strong ion difference of 0 mM [3]. Crystalloids were defined as balanced if they contained weak anions, lowering their chloride content to less than that of normal saline and increasing their in vivo strong ion difference. Studies that used colloids were excluded. See Additional file 1: Table S2 for an overview of the composition of crystalloid solutions from the included studies.

### Outcomes

Our primary outcome was mortality at the longest follow-up. This outcome was selected to be able to include data of potential important studies with a long follow-up time and to generate a common endpoint between studies in an attempt to minimise risk of bias. Secondary outcomes were moderate to severe AKI and the need for new RRT. AKI was defined as Kidney Disease Improving Global Outcomes (KDIGO) stage II or higher, Acute Kidney Injury Network (AKIN) stage II or higher or RIFLE (Risk, Injury, Failure, Loss, End stage) stage Injury or higher. This definition of AKI represents moderate to severe acute kidney injury [20]. See Additional file 1: Table S3 for more details on these AKI classification systems.

### Search strategy

PubMed, EMBASE, Cochrane Library and the WHO International Clinical Trials Registry Platform were searched without language restrictions by three authors (JK, PE, PRT). Databases were searched from inception to April 2019. We used thesaurus terms and free text to define concepts for balanced crystalloids, normal saline and randomized controlled trials and excluded animal studies. The reference lists of included studies and those of review articles were checked to identify other relevant studies. See Additional file 1: Table S4 for detailed search queries.

### Study selection and data extraction

Two authors (PE, PRT) separately screened all retrieved citations by reviewing their titles and abstracts. Then, two reviewers (CLZ, PRT) independently evaluated the full-text manuscripts for eligibility using a standardized form. The same reviewers independently extracted the relevant data. Any disagreements between review authors were resolved by consultation of a third author (PE).

### Risk of bias

Two review authors (CLZ, PRT) independently assessed the study quality, study limitations and the extent of potential bias using the Cochrane Collaboration’s risk of bias tool [21]. The following domains were assessed: sequence generation (selection bias); allocation concealment (selection bias); blinding of participants, personnel and outcome assessors (performance bias and detection bias); incomplete outcome data (attrition bias); selective outcome reporting (reporting bias); baseline characteristics and other potential biases. For each domain, it was judged whether study authors had made sufficient attempts to minimise bias in their study design. Funnel plots for the main outcomes were generated to assess publication bias.

### Meta-analysis

Meta-analyses of our chosen dichotomous outcomes of mortality at latest follow-up, moderate to severe AKI and the need for new RRT were performed using the inverse variance method with random effects model. All analyses were performed separately for the setting of intensive care medicine and emergency medicine. Post hoc subgroup analysis for mortality for patients with sepsis and traumatic brain injury (TBI) was also performed for the setting of intensive care medicine to facilitate comparison with other meta-analysis. Publication bias was assessed by inspection of funnel plots of included studies. Relative risks (RRs) with 95% confidence interval (95% CI) were calculated for all outcomes. *P* values lower than 0.05 were considered to be significant for all analyses. The meta-analyses were performed with RevMan 5 (The Cochrane Collaboration) [22].

### Design factor adjustments

Cluster randomized trials were adjusted for their clustering effects in our meta-analysis. When clustering effects are not taken into account, apparent differences in outcomes between clusters could be magnified and overestimation of the effective sample size can occur as more characteristics and similarities in outcome are shared by patients within clusters when compared with patients between clusters [21, 23]. To adjust for clustering effects, effective sample sizes for cluster randomized trials were approximated by dividing the original sample sizes and event rates by the so called design effect. This design effect was calculated as recommended by the Cochrane Collaboration as follows: design effect = 1 + (*M* − 1) × ICC, where *M* is the average cluster size and ICC is the intracluster correlation coefficient [21]. The ICC is, conceptually, the relative similarity in outcomes of patients within clusters compared to the similarity in outcomes between clusters, or the ratio of between-cluster variance and total variance.

ICC values range from 0 to 1, where small ICCs are indicative of a greater variance within clusters than the variance between clusters [21, 23]. As ICC has not been reported for the included cluster randomized controlled trials, an ICC of 0.011 was used in this meta-analysis based on a previously reported intra-ICU correlation between 35 Australian and New Zealand hospitals [24]. Moreover, a sensitivity analysis was carried out using different imputations (i.e. 0.05, 0.011 and 0.005) for ICC to show its impact on the accrued information sizes (AIS), required information sizes (RIS) and AIS/RIS. An ICC of 0.05 was used in this sensitivity analysis based on the recommendations of the Cochrane Collaboration [21], in which ICCs of 0.05 or lower are recommended. ICC of 0.005 was used to show the impact of even smaller ICCs (Additional file 1: Table S7).

### Quality of evidence

Two authors (CLZ and PRT) independently assessed the quality of evidence generated by this meta-analysis in accordance with the Grading of Recommendations Assessment, Development and Evaluation (GRADE) system [25].

### Trial sequential analysis (TSA)

The risk of random errors of this meta-analysis was assessed with trial sequential analysis [26]. Sequential monitoring boundaries were established to limit the global type I error to 5%. Boundaries were calculated with the O’Brien-Fleming function considering a power of 90% to detect a relative 5% decrease in mortality at the longest follow-up, moderate to severe AKI and the need for new RRT. For baseline mortality rates, we chose the control group mortality for the various settings and subgroups, i.e. baseline mortality rates for mortality at the longest follow-up, moderate to severe AKI and RRT for the setting of intensive care medicine and the emergency department were set at 12.10% and 2.06%, 12.68% and 9.13% and 3.38% and 0.04%, respectively. Baseline mortality rates for septic patients and patients with traumatic brain injury (TBI) were set at 37.95% and 14.14% respectively for the setting of intensive care medicine. The trial sequential analyses were performed with Trial sequential Analysis Viewer (TSAviewer) [Computer Program] 0.9.5.10 Beta (The Copenhagen Trial Unit, Centre for Clinical Intervention Research, Rigshospitalet, Copenhagen, Denmark), 2016.

### Comparisons

From the search results for our meta-analysis, we additionally retrieved published meta-analyses and future trials with a focus on intensive care medicine or emergency medicine. Only English language meta-analyses that were not network meta-analyses and did not include pediatric studies were included. Only planned trials with a target sample size in the order of magnitude of our required information size in our trial sequential analysis were included. From these included meta-analyses and planned studies, we extracted the main results, the alleged information size at the time of analysis and the calculated required information size (RIS) for all subgroups described to directly compare them with our analyses. We evaluated the RIS over a range of assumptions by modeling the RIS on mortality risk, relative risk reduction (RRR) and power (performed in R version 3.5.3).

## Results

The flow diagram describing the study selection process is provided in Additional file 2: Figure S1. Our search strategy identified 1910 references after excluding duplicates. Following screening of titles and abstracts, 88 were selected for full-text assessment. Before resolution, agreement between authors was 91%. From these 88 articles, nine studies were considered for data extraction. Before resolution, agreement between authors was 82%. None of these were unpublished, but one [27] was in abstract format only. Ultimately, all nine trials with a total of 32,777 participants were included in this systematic review. Four of these trials were cluster randomized trials [6, 11, 15, 16], and five were randomized trials at the patient level [5, 7, 8, 27, 28]. Eight of the trials had data available on mortality (*n* = 32,596) [5–8, 11, 15, 16, 28], seven on AKI (*n* = 31,486) [5, 6, 8, 11, 15, 16, 27] and six on the need for new RRT (*n* = 31,612) [6, 8, 11, 15, 16, 27]. Please note that these numbers have not yet been adjusted for design effect.

### Characteristics of included trials and patients

The characteristics of the included studies are listed in Table 1. Studies ranged from 47 to 15,802 patients per study.

**Table 1:** Characteristics of included studies

First author, year	Total number of patients	Setting	Type of balanced crystalloid	Mortality: follow-up period in days	RRT: follow-up period in days	AKI classification (follow-up period in days)	Cumulative volume of fluids in litres, mean ± SD, median (IQR)
ICU-based studies							
Young, 2014 [5]	65	ED and ICU	Plasma-Lyte A	30	–	AKIN (5)	-NS, 9.0 ± 5.5 -Balanced, 10.3 ± 6.5
Young, 2015 [6]	2278	ICU	Plasma-Lyte	In hospital	90	KDIGO ≥ II (90)	-NS, 2.0 (1.0–3.3) -Balanced, 2.0 (1.0–3.5)
Verma, 2016 [8]	70	ICU	Plasma-Lyte	In hospital	In hospital	RIFLE Injury and Failure (4)	-NS, 3.4 (1.2–5.8) -Balanced, 2.9 (1.6–5.6)
Ratanarat, 2017 [27]	181	ICU	Sterofundin	–	–	KDIGO (7)	-NS, 11.2 -Balanced, 11.2
Semler, 2017 [11]	974	ICU	LR or Plasma-Lyte A	60	28	KDIGO ≥ II (30)	-NS, 1.4 (0.5–3.4) -Balanced, 1.6 (0.5–3.6)
Semler, 2018 [15]	15,802	ICU	LR or Plasma-Lyte A	60	28	KDIGO ≥ II (after enrolment)	-NS, 1.02 (0–3.5) -Balanced, 1 (0–3.21)
Included ED-based studies							
Van Zyl, 2012 [7]	51	ED	LR	In hospital	–	–	Not stated
Young, 2014 [5]	65	ED and ICU	Plasma-Lyte A	30	–	AKIN (5)	-NS, 9.0 ± 5.5 -Balanced, 10.3 ± 6.5
Self, 2018 [16]	13,347	ED	LR or Plasma-Lyte A	In hospital	30	KDIGO ≥ II (30)	-NS, 1.07 (1–2) -Balanced, 1.08 (1–2)
Choosakul, 2018 [28]	47	ED	LR	In hospital	–	–	-NS, 5.4 ± 0.8 -Balanced, 4.9 ± 1.3

*ED* emergency department, *ICU* intensive care unit, *LR* lactated Ringer’s, *RRT* renal replacement therapy, *AKI* acute kidney injury, *NS* normal saline, *SD* standard deviation, *IQR* interquartile range

For ICU-based studies, the mean or median cumulative amount of fluid administered to patients ranged from 1 to 11.3 l. Most of the ICU-based studies had a low, 1–3 l study fluid exposure [6, 8, 11, 15]. Aforementioned studies contributed 98.7% (19,124/19370) of the ICU-patients in our meta-analysis. Mean or median volumes of more than 7 l were administered in two ICU-based studies, corresponding with 1.3% (246/19370) of included ICU patients in our meta-analysis [5, 27]. For ED-based studies, the mean or median cumulative amount of fluid administered to patients ranged from 1.07 to 10.3 l. A relatively low fluid exposure of 1–3 l was used in one study, corresponding with the majority of patients in our meta-analysis (98.8%; 13,347/13510) [16]. Mean or median volumes of 4 l or more were administered in 2 trials that contributed 0.8% (112/13510) of the included ED patients [5, 28]. 0.5% (65/13510) of the ED patients in our meta-analysis received mean or median volumes around 7 l or more [5]. One ED-based study, contributing 0.4% (51/13510) of included ED patients did not report the mean or median cumulative amount of study fluid exposure [7]. Please note that these cut-offs (i.e. 1–3, 4 or 7 l) were made to facilitate comparison with previous meta-analyses [13, 17] and have not yet been adjusted for design effect.

### Risk of bias

As illustrated in Additional file 3: Figure S2, the included studies were mostly at low risk of bias. Funnel plots for the main outcomes may be found in Additional file 4: Figure S3. The plot for mortality at the longest follow-up for ICU-based studies was asymmetrical, which implies that publication bias is strongly suspected. No publication bias was evident for moderate to severe AKI or the need for new RRT in ICU-based studies. Based on the small number of included ED-based studies, no definite conclusions on publication bias could be drawn from the corresponding funnel plots. There was no disagreement between authors on the risk of bias.

### Effects on outcome

**Fig. 1:**
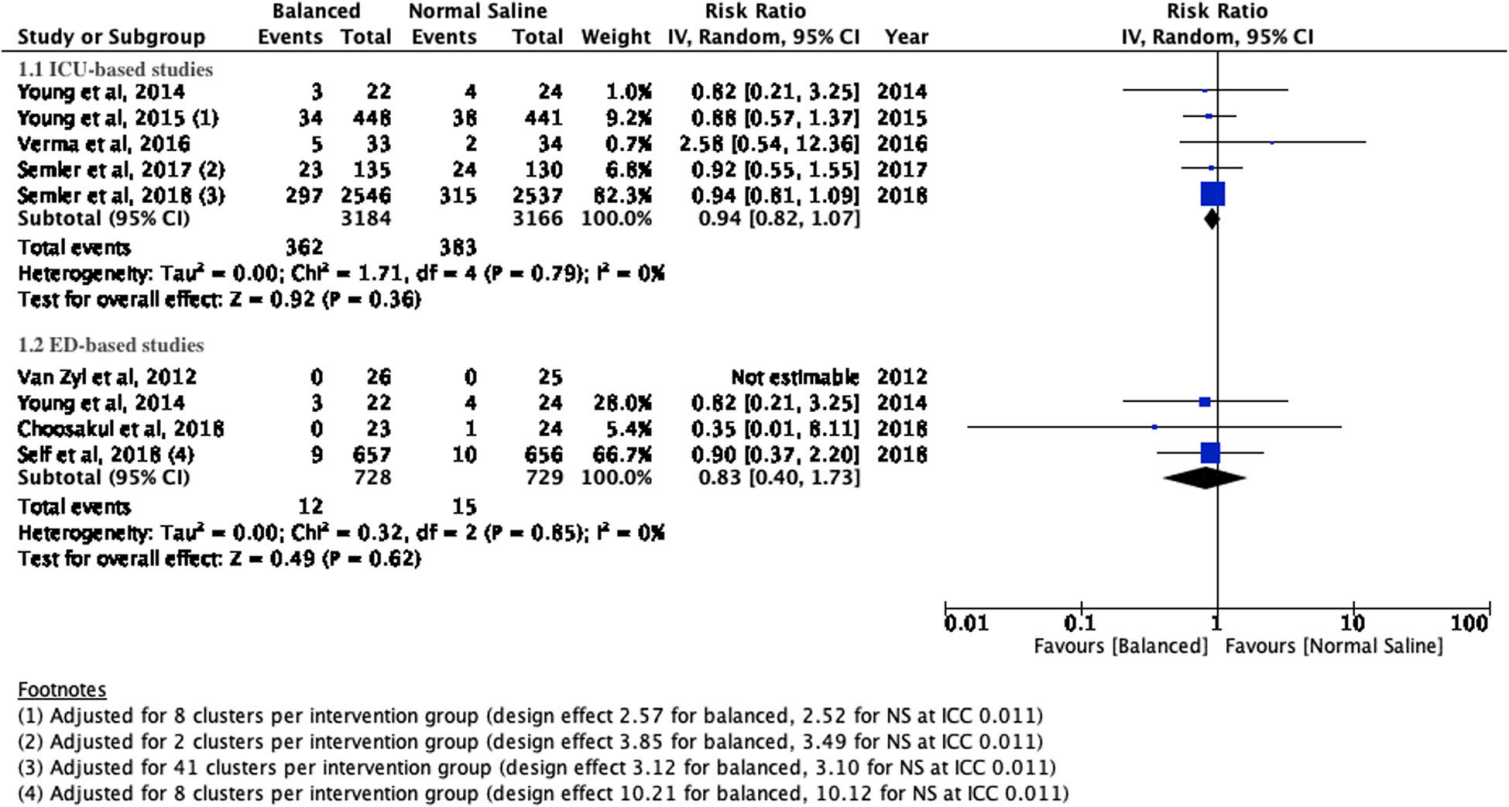
Forest plots for mortality at the longest follow-up for studies performed in the setting of intensive care medicine (1.1) and emergency medicine (1.2)

**Fig. 2:**
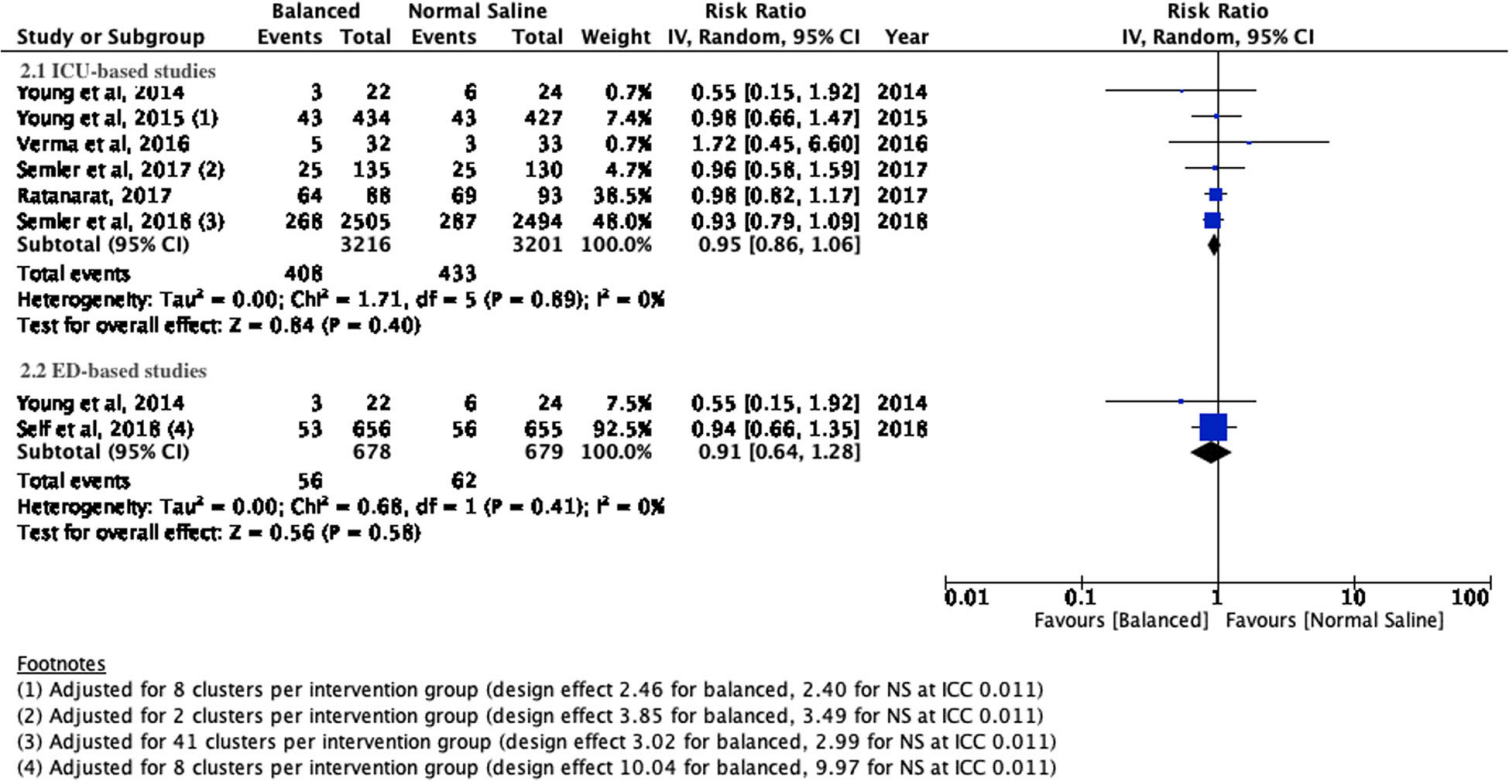
Forest plots for the development of moderate to severe acute kidney injury for studies performed in the setting of intensive care medicine (2.1) and emergency medicine (2.2)

**Fig. 3:**
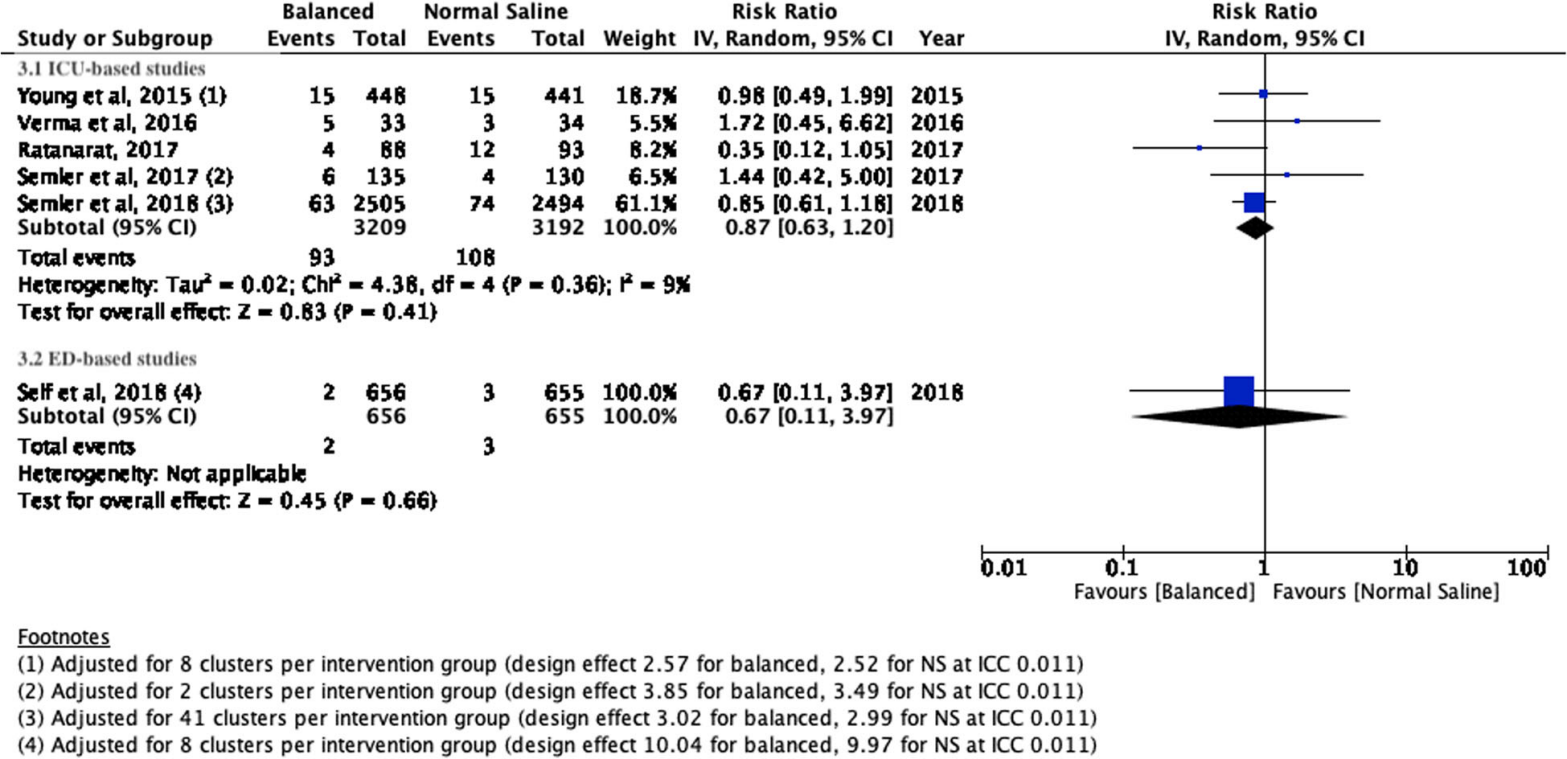
Forest plots for the need of new renal replacement therapy at the longest follow-up for studies performed in the setting of intensive care medicine (3.1) and emergency medicine (3.2)

There were no significant differences for mortality, incidence of moderate or severe AKI or the need for new RRT between patients treated with balanced crystalloids versus normal saline in the setting of the intensive care unit or emergency department. Respectively, the incidences were 11.37% versus 12.10% (ICU; RR 0.94; 95% CI 0.82–1.07; *P* value 0.36) and 1.65 versus 2.06 (ED; RR 0.83; 95% CI 0.40–1.73; *P* value 0.62) for mortality; 12.6% versus 13.53% (ICU; RR 0.95; 95% CI 0.86–1.06; *P* value 0.40) and 8.26% versus 9.13% (ED; RR 0.91; 95% CI 0.64–1.28; *P* value 0.58) for moderate to severe AKI; and 2.89% versus 3.38% (ICU; RR 0.87; 95% CI 0.63–1.20; *P* value 0.41) and 0.30% versus 0.46% (ED; RR 0.67; 95% CI 0.11–3.97; *P* value 0.66) for new RRT. The corresponding Forest plots may be found in Figs. 1, 2 and 3.

Subgroup analysis for mortality for patients with sepsis showed a significant difference between septic patients treated with balanced crystalloids versus normal saline in the setting of intensive care medicine; the incidences were 33.05% (309/935 patients) versus 37.95% (356/938 patients) (RR 0.87; 95% CI 0.77–0.98; *P* value 0.02), respectively. The corresponding Forest plot may be found in Additional file 5: Figure S4.

Subgroup analysis for mortality for patients with TBI showed no significant difference between patients treated with balanced crystalloids versus normal saline in the setting of intensive care medicine (incidences were 15.21% versus 14.14%; RR 1.08; 95% CI 0.82–1.41; *P* value 0.58).

No statistical heterogeneity was detected between balanced crystalloids and normal saline on all outcomes. Yet, clinical heterogeneity was observed in type of balanced crystalloid, cumulative amount of fluid therapy, patient characteristics and follow-up period.

### Quality of evidence

The quality of evidence generated by the meta-analysis as classified according to the GRADE system was very low for mortality and low for moderate to severe AKI and the need for new RRT. See Additional file 1: Table S5 for details. Indirectness was the main reason for downgrading the quality of evidence; in most studies, the cumulative volume of study fluid was low (1–3 l). Studies may be representative for relatively low-risk patients; therefore, high-risk patients who need a moderate to high cumulative volume of fluid were not adequately and directly represented in the majority of studies.

For mortality, another important reason for downgrading evidence quality was the variation in the duration of follow-up. Finally, we downgraded evidence quality for moderate to severe AKI for inconsistency as two studies provided unclear definitions for AKI in their studies [5, 27]. Sensitivity analyses, in which data of the aforementioned studies [5, 27] was excluded, were performed for the development of moderate to severe AKI in the setting of intensive care medicine and emergency medicine. These sensitivity analyses did not alter the results (*P* values of 0.43 and 0.76, respectively) and may be found in Additional file 6: Figure S5. There was no disagreement between authors on the quality of evidence.

### Trial sequential analysis

**Fig. 4:**
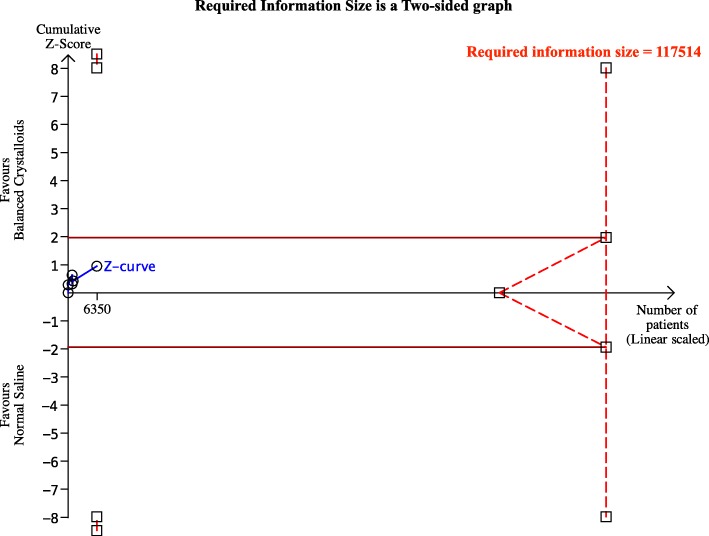
Trial sequential analysis for mortality for the setting of intensive care medicine based on the DerSimonian-Laird random effects model and the O’Brien-Fleming alpha spending function, using estimates of 12.10% for baseline mortality, 5% for relative risk reduction, 5% for alpha and 90% for power. For the setting of emergency medicine, assuming a baseline mortality of 2.06%, no alpha spending boundaries could be calculated because of too small accrued information size

Results of TSA for mortality at the latest follow-up in the setting of intensive care medicine may be found in Fig. 4. Design factor adjustment for sample size was applied for cluster randomized trials. None of the curves crossed the conventional or trial sequential monitoring boundaries for benefit, harm or futility. The required information size for mortality at the latest follow-up for the setting of intensive care medicine was estimated to be 117,514. The accrued information size was 6350. The required information size for mortality at latest follow-up for the setting of intensive care medicine and emergency medicine was estimated to be 827,817. Alpha spending boundaries for trial sequential analyses could not be calculated for mortality in the setting of emergency medicine because of a too small accrued information size (i.e. 1457).

Results of TSA for moderate to severe AKI for the setting of intensive care medicine and for mortality for the subgroup of septic ICU patients may be found in Additional files 7 and 8: Figures S6 and S7. Design factor adjustment for sample size was applied for cluster randomized trials. None of the curves crossed the conventional or trial sequential monitoring boundaries for benefit, harm or futility. The required information size for the secondary outcomes for the setting of intensive care medicine and emergency medicine were estimated to be 114,749 and 161,046 for moderate to severe AKI and 633,156 and 40,962,969 for the need for new RRT, respectively. Alpha spending boundaries for trial sequential analyses could not be calculated for mortality and moderate to severe AKI for the setting of emergency medicine, for mortality for the subgroup of TBI for the setting of intensive care medicine and for RRT for both the setting of intensive care medicine and emergency medicine because of a too small accrued information size.

### Comparisons

**Fig. 5:**
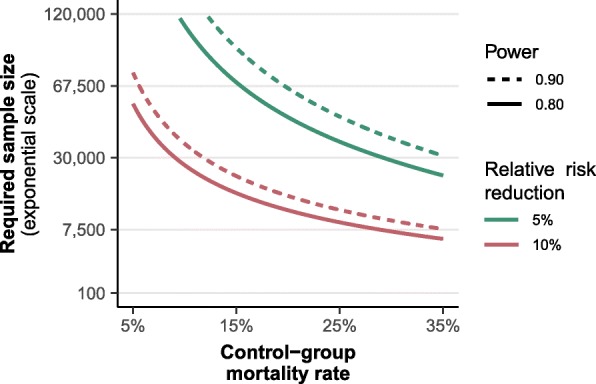
Dependency of sample size on assumptions for baseline mortality risk, power and relative risk reduction

Five meta-analyses [13, 14, 17, 18, 29] and two ongoing trials fulfilled our criteria for extraction. Included studies are given in Additional file 1: Table S6. Results for mortality, accrued and required information sizes and the underlying assumptions may be found in Table 2. For reference, this table also contains that information for the two largest trials that dominated the meta-analyses after their publication. Our meta-analysis adjusted studies for design effect, in contrast to previous meta-analyses, causing the estimated accrued information size to be very different. Additional file 1: Table S7 shows a sensitivity analysis using different imputations (i.e. 0.05, 0.011 and 0.005) for ICC to show its impact on the accrued information size (AIS), required information size (RIS) and AIS/RIS. Besides differences in estimated accrued information size, there were also large differences between observed versus assumed baseline mortality and relative risk reduction for some meta-analyses. Moreover, there were large differences between all meta-analyses and ongoing studies related to assumptions for baseline mortality, relative risk reduction and power, and therefore calculated required information size. Figure 5 illustrates the effect of assumed baseline mortality, relative risk reduction and power on sample size without adjustment for trial diversity.

**Table 2:** Comparison of meta-analyses. Results from meta-analyses before and after the two recent landmark trials on saline versus balanced crystalloids in the setting of intensive care medicine and emergency medicine. For reference, information from two large ongoing trials on this topic has also been included

	Meta-analyses							Current meta-analysis				Landmark trials		Ongoing trials	
First author, year	Serpa, 2017 [29]	Kawano, 2018 [13]	Zayed, 2018 [14]	Liu, 2019 [18]	Xue, 2019 [17]	Xue, 2019 [17]	Xue, 2019 [17]	Zwager	Zwager	Zwager	Zwager	Semler, 2018 [15]	Self, 2018 [16]	BASICS	PLUS
Setting or type of sensitivity analysis	ICU	ICU/OR	ICU	ICU	ICU	Sepsis	TBI	ICU	ED	Sepsis	TBI	ICU	ED	ICU	ICU
*n*	2348	3710	2269	20345	19301	2420	1420	6350	1457	1873	1219	15802	13347	11000	8800
Analysis															
Adjusted for design effect?	No	No	No	No	No	No	No	Yes	Yes	Yes	Yes	–	–	–	–
Mortality (balanced crystalloids, %)	7.54	8.91	11.50	10.33	10.12	24.83	15.21	11.37	1.65	33.05	15.21	11.68	1.40		
Mortality (normal saline, %)	8.57	9.61	12.20	13.17	10.93	28.96	13.77	12.10	2.06	37.95	14.14	12.40	1.54		
Risk ratio	0.88	0.90	0.94	0.93	0.92	0.86	1.11	0.94	0.83	0.87	1.08	0.94	0.90		
*P* value for risk ratio	0.36	0.44	0.10	0.08	0.06	0.02	0.43	0.36	0.62	0.02	0.58	0.10	0.43		
RRR (%)	0.12	0.10	0.06	0.07	0.08	0.14	− 0.11	0.06	0.17	0.13	− 0.08	0.06	0.10		
Assumed baseline mortality (%)		30.00		13.20	10.93	29.00		12.10	2.06	37.95	14.14			35.00	23.00
Assumed RRR (%)		10.00		10.00	6.42	14.48		5.00	5.00	5.00	5.00			10.00	12.50
Alpha (%)		5		5	5	5		5	5	5	5			5	5
Power (%)		80		90	90	90		90	90	90	90			89	90
Required information size (RIS)		9517		26456	80946	4686		117514	827817	27139	99090			11000	8800
Accrued information size (AIS)		3710		20345	19301	2420		6350	1457	1873	1219				
AIS/RIS		0.39		0.77	0.24	0.52		0.05	0.002	0.07	0.01				

*RRR* relative risk reduction, *RIS* required information size, *AIS* accrued information size

## Discussion

Our rigorous meta-analysis with adequate adjustments for design factor for cluster randomized controlled trials could not find differences in mortality at the longest follow-up, the incidence of moderate to severe AKI or the need for new RRT when administering balanced crystalloids or normal saline in the setting of intensive care medicine or emergency medicine. This relegates the findings from the two largest studies to date on the topic favouring balanced crystalloids over normal saline based on improved outcome, albeit on the composite outcome of major adverse kidney events [15, 16].

Having established the current lack of evidence for using balanced fluids over normal saline, it is relevant to assess the feasibility of obtaining such evidence should any difference actually exist. This may be quantified in terms of required information size and the currently accrued information size.

The required information size depends mostly on assumptions regarding baseline outcome rate, relative risk reduction and desired power. So what is a reasonable estimate for the true baseline mortality? Obviously, this depends on disease severity of included patients. For our trial sequential analysis, we chose the control group mortality for the various settings and subgroups. However, it could be argued that a lower value should be chosen to account for publication bias.

And what is a reasonable estimate for the hypothesized relative risk reduction? We chose 5% as a reasonable minimum clinically relevant effect. The true difference might be higher, but given the ubiquity of fluid administration and the small cost differences, it seems reasonable to accept any smaller effect as clinically relevant. Our point assumptions on the baseline outcome rate and the relative treatment effect result in a very high required information size. Figure 5 displays the required sample sizes over a broader range of assumptions.

Regarding the differences in estimations of required information size in previous meta-analysis, it is clear from Table 2 that assumptions on baseline mortality and relative risk vary substantially. To account for publication bias, it is reasonable to assume that the true baseline mortality and relative risk reduction are lower than those found in the meta-analysis. However, none of the previous meta-analysis used mortality rate assumption lower than the rate found in the included studies, and only one previous meta-analysis used a lower relative risk reduction as compared to their findings from the included studies. Would these previous meta-analyses have used more appropriately conservative assumptions, their estimations of required information size would have been much larger.

As for the currently accrued information size, it should be emphasized that all previous trial sequential analyses have not applied adjustments for clustering effects and have therefore grossly overestimated the information size that is currently available. This implies that the number of patients still to be randomized to reach any required information size is equally grossly underestimated. In all, it appears that the available information size has previously been grossly underestimated and that the required information size has likely been grossly overestimated. The effort required for future trials is likely much greater than anticipated.

The ultimate goal of studies on this topic is to better inform the practicing intensivist or emergency physician on what fluid to choose for their patients. In the absence of solid evidence, we cannot ignore possible signal of harm for normal saline from physiology and individual trials [1, 3–10]. On the other hand, preference for balanced solutions with lower sodium also increased incidence of hyponatremia in the SMART study [15]. In addition, the harmful effect of saline remains subject of debate [30–33]. Also, the acetate in some balanced solutions may cause acidosis and hypotension when infusion exceeds metabolic capacity as shown in high flux dialysis [34]. Still, subgroup analysis from the clinical SALT-ED [16] and SMART [15] studies did show that saline seems especially problematic in patients with signs of impaired renal function, in patients with sepsis and those patients receiving large amounts of fluids. In addition, subgroup analysis in this meta-analysis shows a survival benefit for patients with sepsis when balanced fluids are used. Therefore, the burden of proof is currently arguably on normal saline. At the very least, a personalized approach to choosing the right fluid based on physiological reasoning seems advocated.

Within this context and the considerations on required information size, let us now take a closer look at the two large ongoing trials on the topic, BASICS and PLUS. Both studies are carried out in the setting of intensive care medicine. Table 2 shows that for sample size estimation they assumed baseline mortality/relative risk reduction to be 35%/10% and 23%/12% respectively. Although the trialist based these on databases from their previous studies, and these studies aim to include the more severely ill patients of the spectrum, these estimations are arguably high. Thus, their sample size may prove inadequate. But from the perspective of meta-analysis, more problems arise. Should these studies yield an equivocal result or even a result favouring balanced solutions—which in itself would be at risk of a type I error, then their uptake in meta-analysis is not likely to yield conclusive evidence. And if these studies should yield a result favouring normal saline, uptake in meta-analyses will definitely not yield convincing evidence. Importantly, in case of a result favouring one type of fluid over the other, this will only be the case for the more severely ill that will have been included in these trials. This will therefore not silence the debate for patients less severely ill. It is even questionable whether trials are not generally overestimated in this era of personalized medicine [35]. In any case, regardless of the outcome of the two ongoing trials, physiology will continue to guide the choice of fluids in the setting of intensive care medicine and emergency medicine. And currently, physiology favours balanced solutions [36].

Our study has several strengths. First, we took a rigorous approach to all aspects of the analyses, including adjustment for the design factor. We took a conservative approach to estimating the intracluster correlation coefficient as can be concluded from our sensitivity analysis in which different imputations for ICC were used to show its impact on the accrued information size (AIS) and required information size (RIS) (see also Additional file 1: Table S7). Second, we studied both the setting of intensive care medicine and emergency medicine. This is important, as it is obviously challenging to know which patients have elevated creatinine levels on presentation or to predict which patients will progress from mildly ill to being septic and which patients will eventually be needing large amounts of fluids. Surely, intensivists and emergency physicians are unlikely to be willing to conclude that they may not have chosen the most appropriate fluids for their patients in hindsight. Therefore, it may be useful to align standard fluid therapy between the two departments. However, unlike Kawano et al., who pooled studies from the setting of perioperative care and intensive care medicine, we chose to analyse patients treated in the setting of emergency medicine separately from those treated in the setting of intensive care medicine.

We recognize that our study has various limitations. First, the quality of evidence, or absence thereof, was downgraded to very low for mortality and to low for moderate to severe AKI and RRT. This was due to inconsistency, indirectness and publication bias. Moreover, the definition of our main outcome used in the meta-analysis (i.e. mortality ar longest follow up) differed from that registered at PROSPERO (i.e. in-hospital mortality and/or mortality at day 30). A sensitivity analysis using the main outcome registered in PROSPERO was performed, which did not alter the results (see also Additional file 9: Figure S8). We did not include perioperative studies as patients would typically not continue with the assigned fluids upon arrival on the intensive care unit. Also, adding these studies would only minimally increase information size and would risk inclusion of patients that are not acutely or critically ill, as was the case for three earlier meta-analyses [13, 29, 37]. Finally, by definition, meta-analysis suffers from heterogeneity between included studies in terms of patient characteristics and volume administered. The latter may be particularly confounding as possible dosing effects may be masked. Finally, it may be argued that surrogate end points may be preferable to mortality as primary focus. However, all renal outcomes also failed to show a significant difference.

Finally, the vast majority of included studies administered cumulative volumes of IV fluids as low as only 1 to 2 l to patients. Therefore, high-risk patients and more severely ill or septic patients that are more likely to receive moderate to high cumulative fluid volumes may not have been represented adequately. This is relevant given the subgroup analysis from the SMART study showed more pronounced differences in mortality and renal outcomes in septic patients and in those patients that required larger amount of fluids [15].

## Conclusion

Our rigorous meta-analysis could not find a significant different effect on mortality, moderate to severe renal failure or new renal replacement therapy between administering balanced crystalloids or normal saline to patients treated in the setting of intensive care medicine and emergency medicine. In addition, our rigorous trial sequential analysis shows that borders for futility were not crossed, but that the currently accrued information size is smaller and the required information size is much larger than previously anticipated. Therefore, it is strongly debatable whether current ongoing trials on the topic will provide good guidance for practicing intensivists and emergency physicians. This implies that it is likely that the evidence base from physiology, individual trials and observational studies will continue to guide the choice between balanced crystalloids and saline in this setting. This evidence base is limited, but currently arguably places the burden of proof on normal saline and favours balanced crystalloids for the millions of patients treated worldwide with billions of litres of fluids in the setting of intensive care medicine and emergency medicine.

## Supplementary information

**Additional file 1: Table S1.** PRISMA checklist. **Table S2.** Composition of crystalloid solutions in the included studies. **Table S3.** Detailed AKI definitions. **Table S4.** Detailed search strategies. **Table S5.** Quality of evidence. **Table S6.** Included studies in meta-analyses. **Table S7.** Sensitivity analysis for different imputations (i.e. 0.05, 0.011 and 0.005) for intracluster correlation coefficient (ICC) to show its impact on the Accrued Information size (AIS), Required Information Size (RIS) and AIS/RIS.

**Additional file 2: Figure S1.** Flow diagram illustrating the study selection process.

**Additional file 3: Figure S2.** Risk of bias summary.

**Additional file 4: Figure S3.** Funnel plots of included trials.

**Additional file 5: Figure S4.** Forest plots for mortality for patients with sepsis.

**Additional file 6: Figure S5.** Sensitivity analysis for development of moderate to severe acute kidney injury for studies performed in the setting of intensive care medicine (S5.1) and emergency medicine (S5.2).

**Additional file 7: Figure S6.** Trial sequential analysis for moderate to severe AKI for the setting of intensive care medicine based on the DerSimonian-Laird random effects model and the O’Brien-Fleming alpha spending function, using estimates of 12.68% for baseline mortality, 5% for relative risk reduction, 5% for alpha and 90% for power. For the setting of emergency medicine, assuming a baseline incidence of moderate to severe AKI of 9.13%, no alpha spending boundaries could be calculated because of too small accrued information size.

**Additional file 8: Figure S7.** Trial sequential analysis for mortality for patients with sepsis for the setting of intensive care medicine based on the DerSimonian-Laird random effects model and the O’Brien-Fleming alpha spending function, using estimates of 37.95% for baseline mortality, 5% for relative risk reduction, 5% for alpha and 90% for power.

**Additional file 9: Figure S8.** Sensitivity analysis for the outcome mortality using the main outcome registered in PROSPERO (i.e. hospital mortality or 30 day mortality) for studies performed in the setting of intensive care medicine (8.1) and emergency medicine (S8.2)

## Data Availability

All data generated or analysed during this study are included in this published article and its supplementary information files.

## Abbreviations

AKI: Acute kidney injury
RRT: Renal replacement therapy
MAKE30: Major Adverse Kidney Events within 30 days
PRISMA: Preferred reporting items for systematic reviews and meta-analyses
RCT: Randomized controlled trials
NS: Normal saline
KDIGO: Kidney Disease Improving Global Outcomes
AKIN: Acute Kidney Injury Network
RIFLE: Risk, Injury, Failure, Loss, End stage
RR: Relative risks
CI: Confidence interval
DE: Design effect
ICC: Intracluster correlation coefficient
GRADE: Grading of Recommendations Assessment, Development and Evaluation system
TSA: Trial sequential analysis
ICU: Intensive care unit
ED: Emergency department
TBI: Traumatic brain injury
