# Supplementary material for: Why physiology will continue to guide the choice between balanced crystalloids and normal saline: a systematic review and meta-analysis
Source: Crit Care. 2019 Nov 21;23:366. doi: 10.1186/s13054-019-2658-4 (PMC6868741; doi:10.1186/s13054-019-2658-4)
Supplement: Supplementary file 1 — Additional file 1: Table S1. PRISMA checklist. Table S2. Composition of crystalloid solutions in the included studies. Table S3. Detailed AKI definitions. Table S4. Detailed search strategies. Table S5. Quality of evidence. Table S6. Included studies in meta-analyses. Table S7. Sensitivity analysis for different imputations (i.e. 0.05, 0.011 and 0.005) for intracluster correlation coefficient (ICC) to show its impact on the Accrued Information size (AIS), Required Information Size (RIS) and AIS/RIS. [file 13054_2019_2658_MOESM1_ESM.docx]

**Electronic supplementary material**

This appendix has been provided to give readers additional information.

**Supplement to:**

Why physiology will continue to guide the choice between balanced crystalloids and normal saline: a systematic review and meta-analysis by Zwager et al.

**Table S1**. PRISMA checklist.

| **Section/topic** | **#** | **Checklist item** | **Reported on page #** |
| --- | --- | --- | --- |
| **TITLE** |  |  |  |
| Title | 1 | Identify the report as a systematic review, meta-analysis, or both. | 1 |
| **ABSTRACT** |  |  |  |
| Structured summary | 2 | Provide a structured summary including, as applicable: background; objectives; data sources; study eligibility criteria, participants, and interventions; study appraisal and synthesis methods; results; limitations; conclusions and implications of key findings; systematic review registration number. | 2 |
| **INTRODUCTION** |  |  |  |
| Rationale | 3 | Describe the rationale for the review in the context of what is already known. | 3,4 |
| Objectives | 4 | Provide an explicit statement of questions being addressed with reference to participants, interventions, comparisons, outcomes, and study design (PICOS). | 3,4 |
| **METHODS** |  |  |  |
| Protocol and registration | 5 | Indicate if a review protocol exists, if and where it can be accessed (e.g., Web address), and, if available, provide registration information including registration number. | 4 |
| Eligibility criteria | 6 | Specify study characteristics (e.g., PICOS, length of follow-up) and report characteristics (e.g., years considered, language, publication status) used as criteria for eligibility, giving rationale. | 4 |
| Information sources | 7 | Describe all information sources (e.g., databases with dates of coverage, contact with study authors to identify additional studies) in the search and date last searched. | 5, ESM table S4 |
| Search | 8 | Present full electronic search strategy for at least one database, including any limits used, such that it could be repeated. | ESM table S4 |
| Study selection | 9 | State the process for selecting studies (i.e., screening, eligibility, included in systematic review, and, if applicable, included in the meta-analysis). | 5 |
| Data collection process | 10 | Describe method of data extraction from reports (e.g., piloted forms, independently, in duplicate) and any processes for obtaining and confirming data from investigators. | 5 |
| Data items | 11 | List and define all variables for which data were sought (e.g., PICOS, funding sources) and any assumptions and simplifications made. | 5, ESM table S4 |
| Risk of bias in individual studies | 12 | Describe methods used for assessing risk of bias of individual studies (including specification of whether this was done at the study or outcome level), and how this information is to be used in any data synthesis. | 5, ESM Figure S2 |
| Summary measures | 13 | State the principal summary measures (e.g., risk ratio, difference in means). | 6 |
| Synthesis of results | 14 | Describe the methods of handling data and combining results of studies, if done, including measures of consistency (e.g., I^2^) for each meta-analysis. | 6 |
| Risk of bias across studies | 15 | Specify any assessment of risk of bias that may affect the cumulative evidence (e.g., publication bias, selective reporting within studies). | 5,6,ESM |
| Additional analyses | 16 | Describe methods of additional analyses (e.g., sensitivity or subgroup analyses, meta-regression), if done, indicating which were pre-specified. | 5-7 |
| **Results** |  |  |  |
| Study selection | 17 | Give numbers of studies screened, assessed for eligibility, and included in the review, with reasons for exclusions at each stage, ideally with a flow diagram. | 7 |
| Study characteristics | 18 | For each study, present characteristics for which data were extracted (e.g., study size, PICOS, follow-up period) and provide the citations. | 7-8 table 1 |
| Risk of bias within studies | 19 | Present data on risk of bias of each study and, if available, any outcome level assessment (see item 12). | 8, ESM table S3 |
| Results of individual studies | 20 | For all outcomes considered (benefits or harms), present, for each study: (a) simple summary data for each intervention group (b) effect estimates and confidence intervals, ideally with a forest plot. | 8-10, Figures 1-3, ESM FS5 |
| Synthesis of results | 21 | Present results of each meta-analysis done, including confidence intervals and measures of consistency. | 8-10, Figures 1-3, ESM FS5 |
| Risk of bias across studies | 22 | Present results of any assessment of risk of bias across studies (see Item 15). | 9, ESM table S5 |
| Additional analysis | 23 | Give results of additional analyses, if done (e.g., sensitivity or subgroup analyses, meta-regression [see Item 16]). | 9-10, table 2, ESM table S6 |
| **DISCUSSION** |  |  |  |
| Summary of evidence | 24 | Summarize the main findings including the strength of evidence for each main outcome; consider their relevance to key groups (e.g., healthcare providers, users, and policy makers). | 10-13 |
| Limitations | 25 | Discuss limitations at study and outcome level (e.g., risk of bias), and at review-level (e.g., incomplete retrieval of identified research, reporting bias). | 10-13 |
| Conclusions | 26 | Provide a general interpretation of the results in the context of other evidence, and implications for future research. | 13 |
| **FUNDING** |  |  |  |
| Funding | 27 | Describe sources of funding for the systematic review and other support (e.g., supply of data); role of funders for the systematic review. | 20 |

**Table S2**. Composition of crystalloid solutions in the included studies.

|  | Human Plasma | Normal Saline | Lactated Ringer's | Plasma-Lyte A | Sterofundin |
| --- | --- | --- | --- | --- | --- |
| Sodium (mmol/L) | 136-145 | 154 | 130 | 140 | 145 |
| Potassium (mmol/L) | 3.5-5 | - | 4 | 5 | 4 |
| Chloride (mmol/L) | 94-106 | 154 | 109 | 98 | 127 |
| Calcium (mmol/L) | 2.2–2.6 | - | 3 | - | 2.5 |
| Magnesium (mmol/L) | 0.8–1 | - |  | 1.5 | 1 |
| Lactate (mmmol/L) |  |  | 28 |  | 1.5 |
| Acetate (mmol/L) |  |  |  | 27 | 24 |
| Gluconate (mmol/L) |  |  |  | 23 |  |
| Malate (mmol/L) |  |  |  |  | 5 |
| SID (mEq/L) | 42 | 0 | 28 | 50 | 29 |

SID: strong ion difference

**Table S3**. Detailed AKI definitions.

|  | **RIFLE Stage Risk** | **KDIGO Stage I** | **AKIN Stage I** |
| --- | --- | --- | --- |
| Creatinin increase | >50%  or >0.3 mg/dL | >50% | |
| Urine output | <0.5 ml/kg for 6-12 h | | |

|  | **RIFLE Stage Injury** | **KDIGO  Stage II** | **AKIN Stage II** |
| --- | --- | --- | --- |
| Creatinine increase | >100% | >100% | >100% |
| Urine output | <0.5 ml/kg for 6-12 h | | |

|  | **RIFLE Stage Failure** | **KDIGO  Stage III** | **AKIN Stage III** |
| --- | --- | --- | --- |
| Creatinine increase | >200%  or >0.5 mg/dL to >4.0 mg/dL | | >200%  or >0.3 mg/dL to >4.0 mg/dL |
| Urine output | <0.3 mL/kg for >24 hours  or anuria for >12 hours  or initiation of RRT | | |

**Table S4**. Detailed search strategies.

**Source: Pubmed, 3th of April 2019**

| **Search** | **Query** |
| --- | --- |
| #26 | (#25 NOT (animals[mh] NOT humans[mh])) |
| #25 | (#21 AND #22 AND (#23 OR #24)) |
| #24 | (((((random*[tiab] AND (controlled[tiab] OR control[tiab] OR placebo[tiab] OR versus[tiab] OR vs[tiab] OR group[tiab] OR groups[tiab] OR comparison[tiab] OR compared[tiab] OR arm[tiab] OR arms[tiab] OR crossover[tiab] OR cross-over[tiab]) AND (trial[tiab] OR study[tiab])) OR ((single[tiab] OR double[tiab] OR triple[tiab]) AND (masked[tiab] OR blind*[tiab])))))) |
| #23 | (((((review*[tiab] OR search*[tiab] OR survey*[tiab] OR handsearch*[tiab] OR hand-search*[tiab]) AND (databa*[tiab] OR data-ba*[tiab] OR bibliograph*[tiab] OR electronic*[tiab] OR medline*[tiab] OR pubmed*[tiab] OR embase*[tiab] OR Cochrane[tiab] OR cinahl[tiab] OR psycinfo[tiab] OR psychinfo[tiab] OR cinhal[tiab] OR "web of science"[tiab] OR "web of knowledge"[tiab] OR ebsco[tiab] OR ovid[tiab] OR mrct[tiab] OR metaregist*[tiab] OR meta-regist*[tiab] OR ((predetermined[tiab] OR pre-determined[tiab]) AND criteri*[tiab]) OR apprais*[tiab] OR inclusion criteri*[tiab] OR exclusion criteri*[tiab])) OR (review[pt] AND systemat*[tiab]) OR "systematic review"[tiab] OR "systematic literature"[tiab] OR "integrative review"[tiab] OR "integrative literature"[tiab] OR "evidence-based review"[tiab] OR "evidence-based overview"[tiab] OR "evidence-based literature"[tiab] OR "evidence-based survey"[tiab] OR "literature search"[tiab] OR ((systemat*[ti] OR evidence-based[ti]) AND (review*[ti] OR literature[ti] OR overview[ti] OR survey[ti])) OR "data synthesis"[tiab] OR "evidence synthesis"[tiab] OR "data extraction"[tiab] OR "data source"[tiab] OR "data sources"[tiab] OR "study selection"[tiab] OR "methodological quality"[tiab] OR "methodologic quality"[tiab] OR cochrane database syst rev[ta] OR meta-analy*[tiab] OR metaanaly*[tiab] OR metanaly*[tiab] OR meta-analysis[pt] OR meta-synthesis[tiab] OR metasynthesis[tiab] OR meta-study[tiab] OR metastudy[tiab] OR metaethnograph*[tiab] OR meta-ethnograph*[tiab] OR Technology Assessment, Biomedical[mh] OR hta[tiab] OR health technol assess [ta] OR evid rep technol assess summ[ta] OR health technology assessment[tiab]) OR ((review*[ot] OR search*[ot] OR survey*[ot] OR handsearch*[ot] OR hand-search*[ot]) AND (databa*[ot] OR data-ba*[ot] OR bibliograph*[ot] OR electronic*[ot] OR medline*[ot] OR pubmed*[ot] OR embase*[ot] OR cochrane[ot] OR cinahl[ot] OR psycinfo[ot] OR psychinfo[ot] OR cinhal[ot] OR "web of science"[ot] OR "web of knowledge"[ot] OR ebsco[ot] OR ovid[ot] OR mrct[ot] OR metaregist*[ot] OR meta-regist*[ot] OR ((predetermined[ot] OR pre-determined[ot]) AND criteri*[ot]) OR apprais*[ot] OR inclusion criteri*[ot] OR exclusion criteri*[ot]) OR (review[pt] AND systemat*[ot]) OR "systematic review"[ot] OR "systematic literature"[ot] OR "integrative review"[ot] OR "integrative literature"[ot] OR "evidence-based review"[ot] OR "evidence-based overview"[ot] OR "evidence-based literature"[ot] OR "evidence-based survey"[ot] OR "literature search"[ot] OR ((systemat*[ti] OR evidence-based[ti]) AND (review*[ti] OR literature[ti] OR overview[ti] OR survey[ti])) OR "data synthesis"[ot] OR "evidence synthesis"[ot] OR "data extraction"[ot] OR "data source"[ot] OR "data sources"[ot] OR "study selection"[ot] OR "methodological quality"[ot] OR "methodologic quality"[ot] OR meta-analy*[ot] OR metaanaly*[ot] OR metanaly*[ot] OR meta-analysis[pt] OR meta-synthesis[ot] OR metasynthesis[ot] OR meta-study[ot] OR metastudy[ot] OR metaethnograph*[ot] OR meta-ethnograph*[ot] OR hta[ot] OR health technology assessment[ot])))) |
| #22 | ((("Sodium Chloride"[Mesh] OR saline[tiab] OR nacl*[tiab] OR sodium chlorid*[tiab] OR salt[tiab] OR (high[tiab] AND chlorid*[tiab])))) |
| #21 | (((((balance*[ti] OR buffer*[ti]) AND ("Solutions"[Mesh] OR "crystalloid solutions" [Supplementary Concept] OR "Infusions, Intravenous"[Mesh] OR infusion*[ti] OR infusat*[ti] OR intravenous*[ti] OR crystalloid*[ti] OR fluid*[ti] OR solution*[ti] OR lactate*[ti] OR acetate*[ti] OR "Sodium Chloride"[Mesh] OR saline[ti] OR nacl*[ti] OR sodium chlorid*[ti])) OR "Plasmalyte A" [Supplementary Concept] OR "Plasma-lyte 148"[Supplementary Concept] OR plasma lyt*[ti] OR plasmalyt*[ti] OR (low[ti] AND chlorid*[ti]) OR ionolyt*[ti] OR jonolyt*[ti] OR sterofundin*[ti] OR isofundin*[ti] OR isolyte*[ti] OR normosol*[ti] OR plasmasol*[ti] OR ringerfundin*[ti] OR ((balance*[ot] OR buffer*[ot]) AND (crystalloid*[ot] OR infusion*[ot] OR infusat*[ot] OR intravenous*[ot] OR fluid*[ot] OR solution*[ot] OR lactate*[ot] OR acetate*[ot] OR saline[ot] OR nacl*[ot] OR sodium chlorid*[ot])) OR plasma lyt*[ot] OR plasmalyt*[ot] OR (low[ot] AND chlorid*[ot]) OR ionolyt*[ot] OR jonolyt*[ot] OR sterofundin*[ot] OR isofundin*[ot] OR isolyte*[ot] OR normosol*[ot] OR plasmasol*[ot] OR ringerfundin*[ot]) OR ("Ringer's lactate"[Supplementary Concept] OR "Krebs-Ringer HEPES bicarbonate solution"[Supplementary Concept] OR "Krebs-Ringer solution"[Supplementary Concept] OR "Ascaris Ringers solution"[Supplementary Concept] OR krebs ringer*[tiab] OR ringers[tiab] OR ringer[tiab] OR ringer's[tiab] OR hartmann*[tiab] OR hartman*[tiab] OR krh solution*[tiab] OR ars solution*[tiab]))) |

**Source: Embase.com, 3th of April, 2019**

| No. | Query |
| --- | --- |
| #16 | #15 NOT [373 PMIDs] |
| #15 | #14 NOT [medline]/lim |
| #14 | #13 NOT ('conference abstract'/it OR 'conference paper'/it OR 'conference review'/it OR 'erratum'/it OR 'letter'/it) |
| #13 | #12 NOT ([animals]/lim NOT [humans]/lim) |
| #12 | #9 AND (#10 OR #11) |
| #11 | ('meta analysis'/exp OR 'systematic review'/exp OR ((meta NEAR/3 analy*):ab,ti) OR metaanaly*:ab,ti OR review*:ti OR overview*:ti OR ((synthes* NEAR/3 (literature* OR research* OR studies OR data)):ab,ti) OR (pooled AND analys*:ab,ti) OR (((data NEAR/2 pool*):ab,ti) AND studies:ab,ti) OR medline:ab,ti OR medlars:ab,ti OR embase:ab,ti OR cinahl:ab,ti OR scisearch:ab,ti OR psychinfo:ab,ti OR psycinfo:ab,ti OR psychlit:ab,ti OR psyclit:ab,ti OR cinhal:ab,ti OR cancerlit:ab,ti OR cochrane:ab,ti OR bids:ab,ti OR pubmed:ab,ti OR ovid:ab,ti OR (((hand OR manual OR database* OR computer*) NEAR/2 search*):ab,ti) OR ((electronic NEAR/2 (database* OR 'data base' OR 'data bases')):ab,ti) OR bibliograph*:ab OR 'relevant journals':ab OR (((review* OR overview*) NEAR/10 (systematic* OR methodologic* OR quantitativ* OR research* OR literature* OR studies OR trial* OR effective*)):ab)) NOT ((((retrospective* OR record* OR case* OR patient*) NEAR/2 review*):ab,ti) OR (((patient* OR review*) NEAR/2 chart*):ab,ti)) NOT ('editorial'/exp OR 'erratum'/de OR 'letter'/exp) |
| #10 | random*:ti,ab,kw OR factorial*:ti,ab,kw OR crossover*:ti,ab,kw OR ((cross NEXT/1 over*):ti,ab,kw) OR placebo*:ti,ab,kw OR (doubl*:ti,ab,kw AND blind*:ti,ab,kw) OR (singl*:ti,ab,kw AND blind*:ti,ab,kw) OR assign*:ti,ab,kw OR allocat*:ti,ab,kw OR volunteer*:ti,ab,kw OR 'crossover procedure'/exp OR 'double blind procedure'/exp OR 'randomized controlled trial'/exp OR 'single blind procedure'/exp |
| #9 | #7 AND #8 |
| #8 | 'sodium chloride'/exp OR saline:ti,ab,kw OR nacl*:ti,ab,kw OR 'sodium chlorid*':ti,ab,kw OR salt:ti,ab,kw OR ((high NEAR/3 chlorid*):ti,ab,kw) |
| #7 | #5 OR #6 |
| #6 | 'acetic acid plus gluconate sodium plus magnesium chloride plus potassium chloride plus sodium chloride'/exp OR 'plasma lyt*':ti,kw OR plasmalyt*:ti,kw OR ((low NEAR/3 chlorid*):ti,kw) OR ionolyt*:ti,kw OR jonolyt*:ti,kw OR sterofundin*:ti,kw OR isofundin*:ti,kw OR isolyte*:ti,kw OR normosol*:ti,kw OR plasmasol*:ti,kw OR ringerfundin*:ti,kw OR 'calcium chloride plus potassium chloride plus sodium chloride'/exp OR 'krebs ringer*':ti,ab,kw OR ringers:ti,ab,kw OR ringer:ti,ab,kw OR 'ringer s':ti,ab,kw OR hartmann*:ti,ab,kw OR hartman*:ti,ab,kw OR 'krh solution*':ti,ab,kw OR 'ars solution*':ti,ab,kw |
| #5 | #1 OR #4 |
| #4 | #2 AND #3 |
| #3 | 'infusion fluid'/exp OR 'solution and solubility'/exp OR 'crystalloid'/exp OR 'intravenous drug administration'/exp OR infusion*:ti,kw OR infusat*:ti,kw OR intravenous*:ti,kw OR crystalloid*:ti,kw OR fluid*:ti,kw OR solution*:ti,kw OR lactate*:ti,kw OR acetate*:ti,kw OR 'sodium chloride'/exp OR saline:ti,kw OR nacl*:ti,kw OR 'sodium chlorid*':ti,kw |
| #2 | balance*:ti,kw OR buffer*:ti,kw OR 'buffer'/exp |
| #1 | 'balanced salt solution'/exp |

**Source: Wiley/Cochrane Library 3th of April, 2019**

| ID | Search |
| --- | --- |
| #1 | (((balance* or buffer*) near/3 (infusion* or infusat* or intravenous* or crystalloid* or fluid* or solution* or lactate* or acetate* or saline or nacl* or "sodium chlorid*")) or "plasma lyt*" or plasmalyt* or (low near/3 chlorid*) or ionolyt* or jonolyt* or sterofundin* or isofundin* or isolyte* or normosol* or plasmasol* or ringerfundin* or "krebs ringer*" or ringers or ringer or "ringer s" or hartmann* or hartman* or "krh solution*" or "ars solution*"):ti,kw |
| #2 | (saline or nacl* or "sodium chlorid*" or salt or (high near/3 chlorid*)):ti,kw |
| #3 | #1 AND #2 |

**Source: WHO/ICTRP 3 apr 2019 (258)**

TITLE

balance* AND infusion* OR balance* AND infusat* OR balance* AND intravenous* OR balance* AND crystalloid* OR balance* AND fluid* OR balance* AND solution* OR balance* AND lactate* OR balance* AND acetate* OR balance* AND saline OR balance* AND nacl* OR balance* AND sodium chlorid* - 59

buffer* AND infusion* OR buffer* AND infusat* OR buffer* AND intravenous* OR buffer* AND crystalloid* OR buffer* AND fluid* OR buffer* AND solution* OR buffer* AND lactate* OR buffer* AND acetate* OR buffer* AND saline OR buffer* AND nacl* OR buffer* AND sodium chlorid* - 5

plasma lyt* OR plasmalyt* OR (low AND chlorid*) OR ionolyt* OR jonolyt* OR sterofundin* OR isofundin* OR isolyte* OR normosol* OR plasmasol* OR ringerfundin* OR krebs ringer* OR ringers OR ringer OR ringer s OR hartmann* OR hartman* OR krh solution* OR ars solution* - 194

**Table S5**. Quality of evidence.

| **Outcome** | **Quality Assessment** | | | | | | **Quality of evidence** |
| --- | --- | --- | --- | --- | --- | --- | --- |
|  | **Study design** | **Risk of bias** | **Inconsistency** | **Indirectness** | **Imprecision** | **Publication bias** |  |
| In-hospital mortality:  8 studies included  (32596 patients) | No serious limitations | No serious risk of bias | Inconsistency (-1) ^a^ | Indirectness  (-1) ^b^ | No serious imprecision | Publication bias was strongly suspected ^c^ | ⊕⊝⊝⊝ Very low |
| AKI:  7 studies included  (31486 patients) | No serious limitations | No serious risk of bias | Inconsistency (-1) ^a^ | Indirectness  (-1) ^b^ | No serious imprecision | Unlikely | ⊕⊕⊝⊝ Low |
| RRT:  6 studies included  (31612 patients) | No serious limitations | No serious risk of bias | Inconsistency (-1) ^a^ | Indirectness  (-1) ^b^ | No serious imprecision | Unlikely | ⊕⊕⊝⊝ Low |

^a^ Statistical heterogeneity was low (I^2^=0). But clinical heterogeneity between studies was detected.

^b^ The majority of the studies administered a low dose of crystalloids (1-3L). Studies may be representative for relatively low-risk patients, therefore, high risk patients who need a moderate or high cumulative volume of fluid were not adequately and directly represented in the majority of studies.

^c^ Funnel plot was asymmetrical, indicative of publication bias.

**Table S6**. Included studies in meta-analyses.

**Table S7.** Sensitivity analysis for different imputations (i.e. 0.05, 0.011 and 0.005) for intracluster correlation coefficient (ICC) to show its impact on the Accrued Information size (AIS), Required Information Size (RIS) and AIS/RIS.

|  | **Mortality for ICU-based studies** | | | **Mortality for ED-based studies** | | |
| --- | --- | --- | --- | --- | --- | --- |
| ICC | 0.005 | 0.011 | 0.05 | 0.005 | 0.011 | 0.05 |
| n (adjusted for design effect) | 9950 | 6350 | 1962 | 5005 | 1457 | 456 |
|  |  |  |  |  |  |  |
| Assumed baseline mortality (%) | 12.10 | 12.10 | 12.10 | 2.06 | 2.06 | 2.06 |
| Assumed RRR (%) | 5.00 | 5.00 | 5.00 | 5.00 | 5.00 | 5.00 |
| Alpha (%) | 5 | 5 | 5 | 5 | 5 | 5 |
| Power (%) | 90 | 90 | 90 | 90 | 90 | 90 |
|  |  |  |  |  |  |  |
| Required Information Size (RIS) | 117514 | 117514 | 117514 | 827817 | 827817 | 827817 |
| Accrued Information Size (AIS) | 9950 | 6350 | 1962 | 5005 | 1457 | 456 |
| AIS/RIS | 0.08 | 0.05 | 0.02 | 0.006 | 0.002 | 0.0006 |

ICU: intensive care unit; ED: emergency department; ICC: intracluster correlation coefficient; RRR: Relative Risk Reduction RIS: Required Information Size; AIS: Accrued Information Size.

**Figure Captions**

Figure S1. Flow diagram illustrating the study selection process.

Figure S2. Risk of bias summary.

Figure S3. Funnel plots of included trials.

Figure S4. Forest plots for mortality for patients with sepsis.

Figure S5. Sensitivity analysis for development of moderate to severe acute kidney injury for studies performed in the setting of intensive care medicine (S5.1) and emergency medicine (S5.2).

Figure S6. Trial sequential analysis for moderate to severe AKI for the setting of intensive care medicine based on the DerSimonian-Laird random effects model and the O’Brien-Fleming alpha spending function, using estimates of 12.68 % for baseline mortality, 5% for relative risk reduction, 5% for alpha and 90% for power. For the setting of emergency medicine, assuming a baseline incidence of moderate to severe AKI of 9.13 %, no alpha spending boundaries could be calculated because of too small accrued information size.

Figure S7. Trial sequential analysis for mortality for patients with sepsis for the setting of intensive care medicine based on the DerSimonian-Laird random effects model and the O’Brien-Fleming alpha spending function, using estimates of 37.95 % for baseline mortality, 5% for relative risk reduction, 5% for alpha and 90% for power.

Figure S8. Sensitivity analysis for the outcome mortality using the main outcome registered in PROSPERO (i.e. hospital mortality or 30 day mortality) for studies performed in the setting of intensive care medicine (S8.1)) and emergency medicine (S8.2).
