# Supplementary figures and images for: Why physiology will continue to guide the choice between balanced crystalloids and normal saline: a systematic review and meta-analysis
Source: Crit Care. 2019 Nov 21;23:366. doi: 10.1186/s13054-019-2658-4 (PMC6868741; doi:10.1186/s13054-019-2658-4)

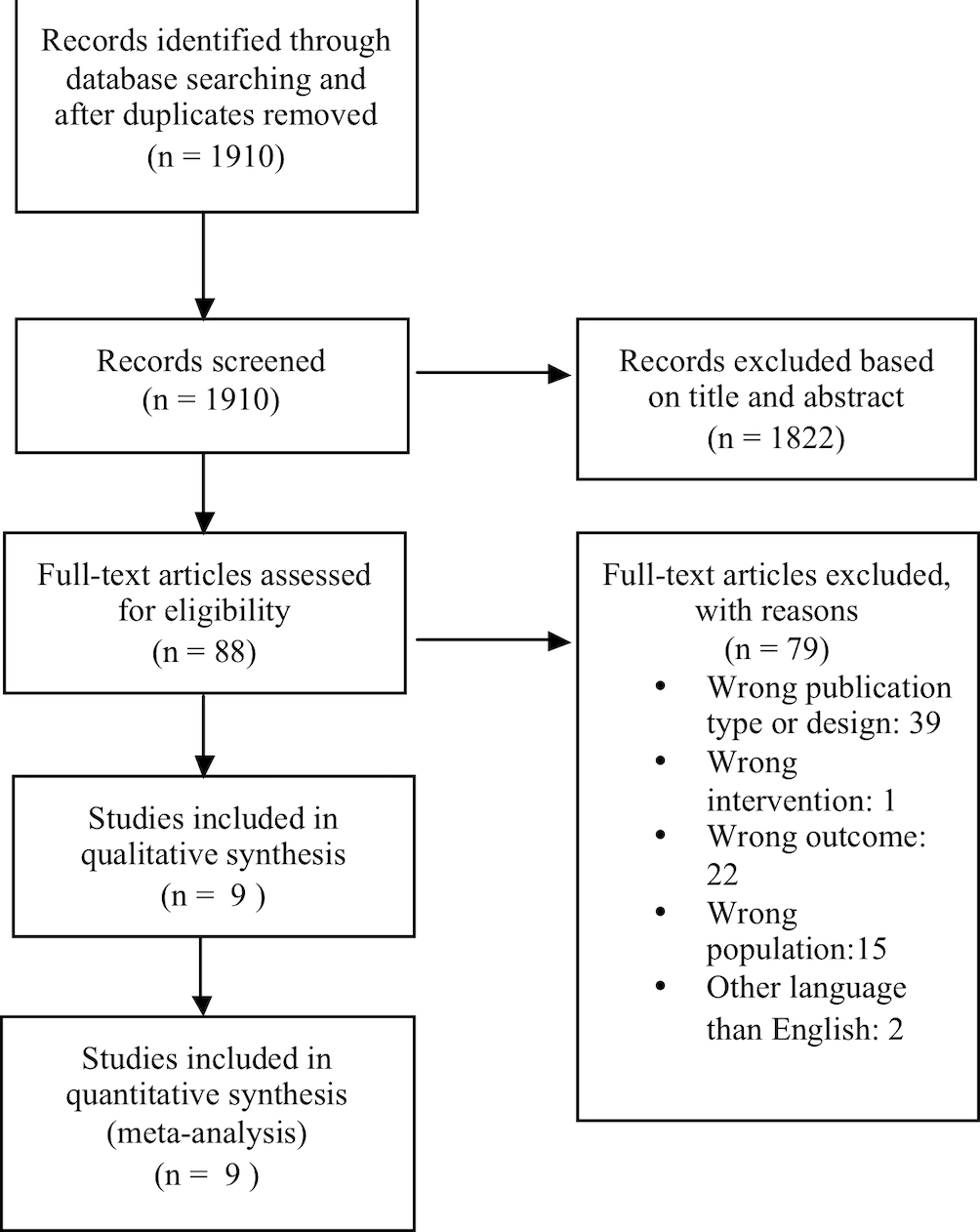

Supplement: Supplementary file 2 — Additional file 2: Figure S1. Flow diagram illustrating the study selection process. [file 13054_2019_2658_MOESM2_ESM.png]

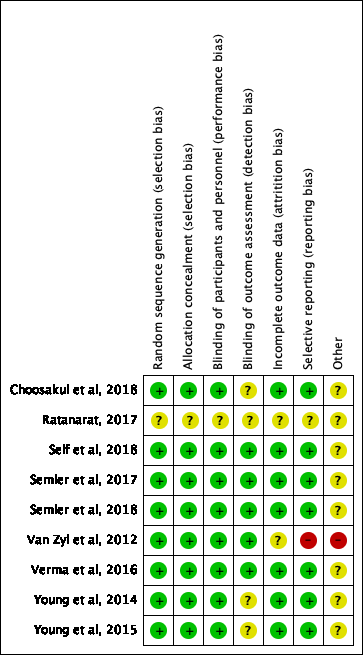

Supplement: Supplementary file 3 — Additional file 3: Figure S2. Risk of bias summary. [file 13054_2019_2658_MOESM3_ESM.png]

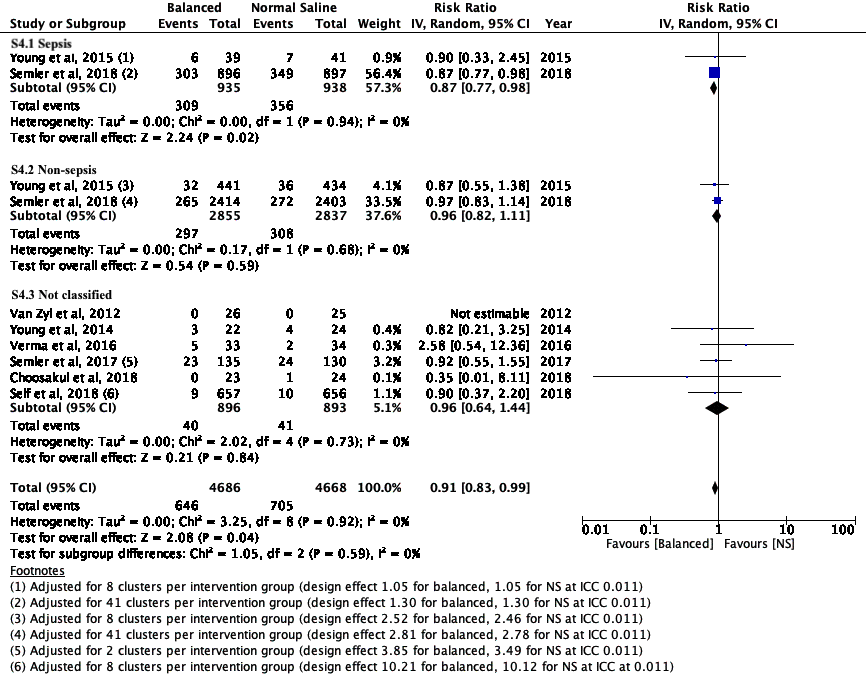

Supplement: Supplementary file 5 — Additional file 5: Figure S4. Forest plots for mortality for patients with sepsis. [file 13054_2019_2658_MOESM5_ESM.png]

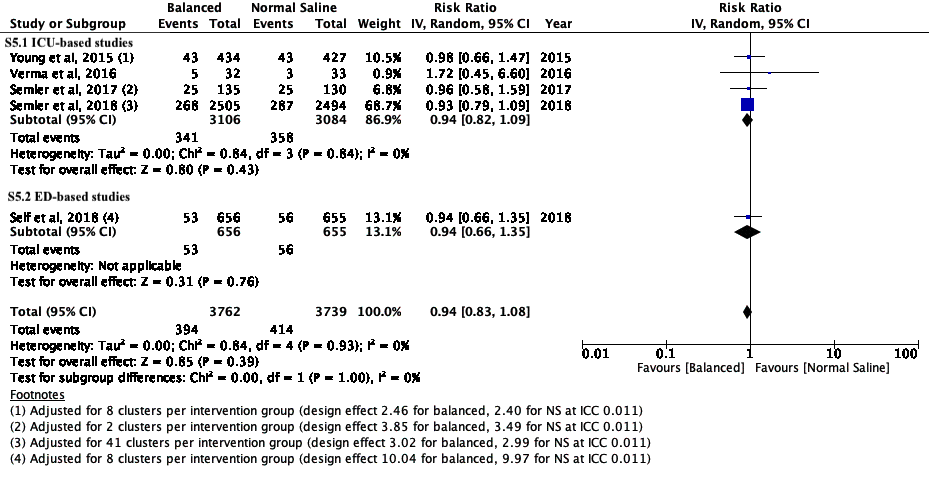

Supplement: Supplementary file 6 — Additional file 6: Figure S5. Sensitivity analysis for development of moderate to severe acute kidney injury for studies performed in the setting of intensive care medicine (S5.1) and emergency medicine (S5.2). [file 13054_2019_2658_MOESM6_ESM.png]

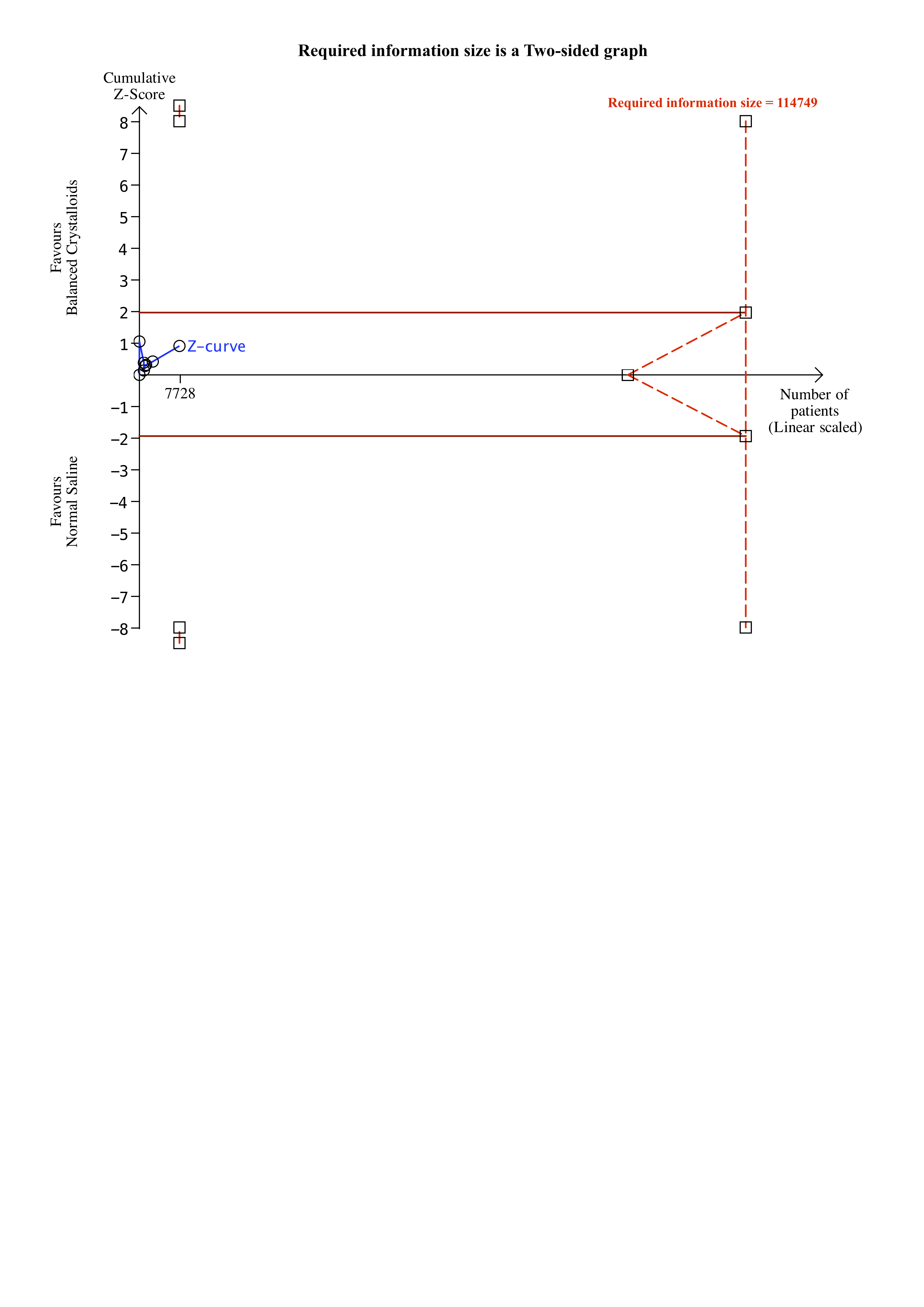

Supplement: Supplementary file 7 — Additional file 7: Figure S6. Trial sequential analysis for moderate to severe AKI for the setting of intensive care medicine based on the DerSimonian-Laird random effects model and the O’Brien-Fleming alpha spending function, using estimates of 12.68% for baseline mortality, 5% for relative risk reduction, 5% for alpha and 90% for power. For the setting of emergency medicine, assuming a baseline incidence of moderate to severe AKI of 9.13%, no alpha spending boundaries could be calculated because of too small accrued information size. [file 13054_2019_2658_MOESM7_ESM.png]

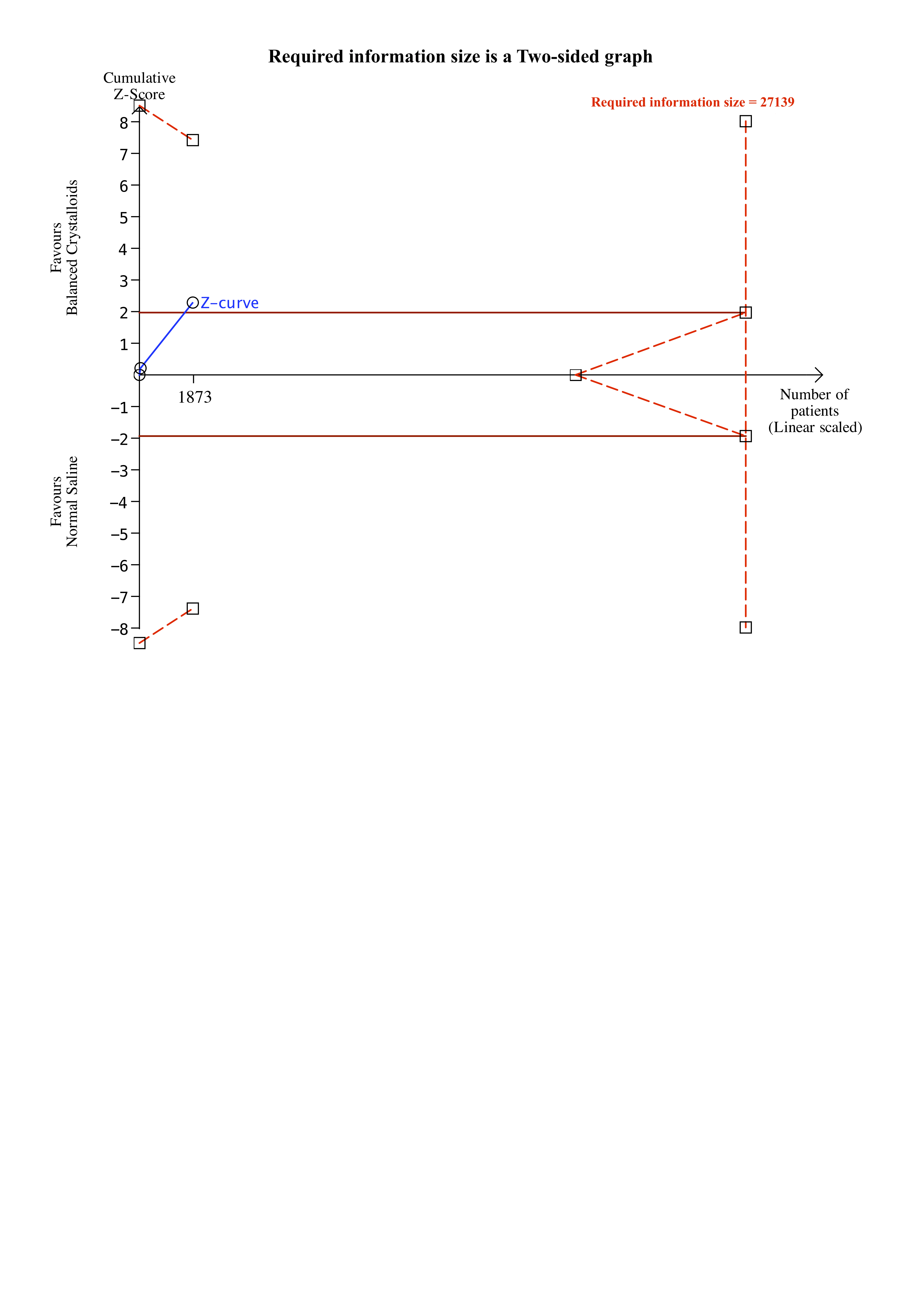

Supplement: Supplementary file 8 — Additional file 8: Figure S7. Trial sequential analysis for mortality for patients with sepsis for the setting of intensive care medicine based on the DerSimonian-Laird random effects model and the O’Brien-Fleming alpha spending function, using estimates of 37.95% for baseline mortality, 5% for relative risk reduction, 5% for alpha and 90% for power. [file 13054_2019_2658_MOESM8_ESM.png]

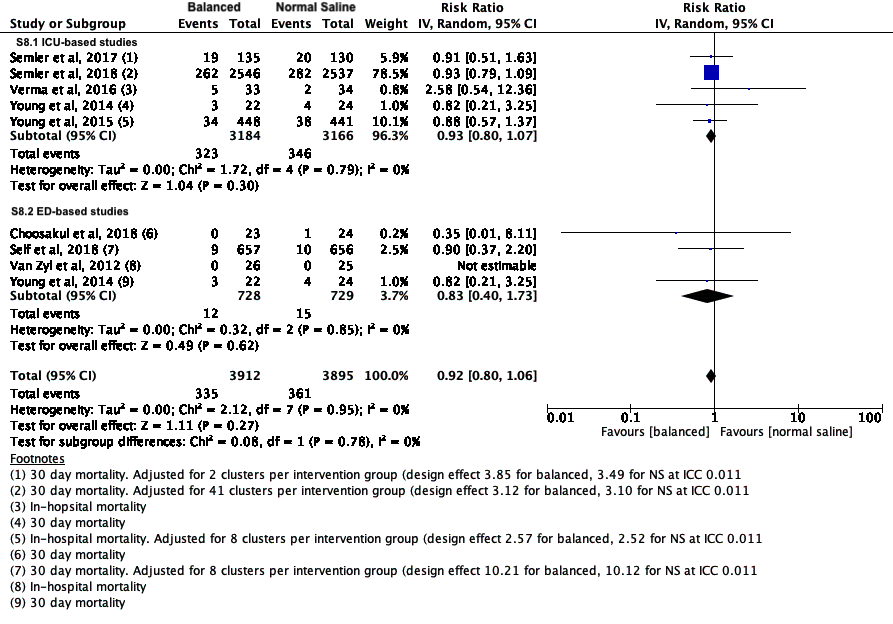

Supplement: Supplementary file 9 — Additional file 9: Figure S8. Sensitivity analysis for the outcome mortality using the main outcome registered in PROSPERO (i.e. hospital mortality or 30 day mortality) for studies performed in the setting of intensive care medicine (8.1) and emergency medicine (S8.2) [file 13054_2019_2658_MOESM9_ESM.png]
