# Supplementary material for: Why physiology will continue to guide the choice between balanced crystalloids and normal saline: a systematic review and meta-analysis
Source: Crit Care. 2019 Nov 21;23:366. doi: 10.1186/s13054-019-2658-4 (PMC6868741; doi:10.1186/s13054-019-2658-4)

S3.1 Funnel plot of mortality at longest follow up for ICU-based studies

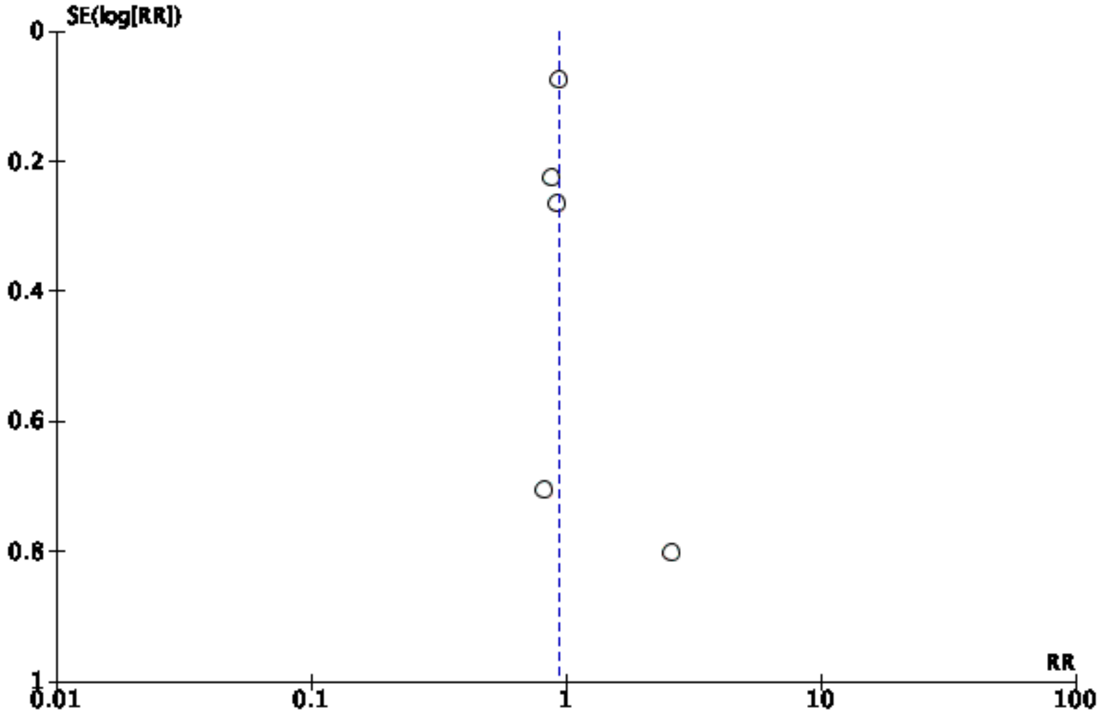

S3.2 Funnel plot of mortality at longest follow up for ED-based studies

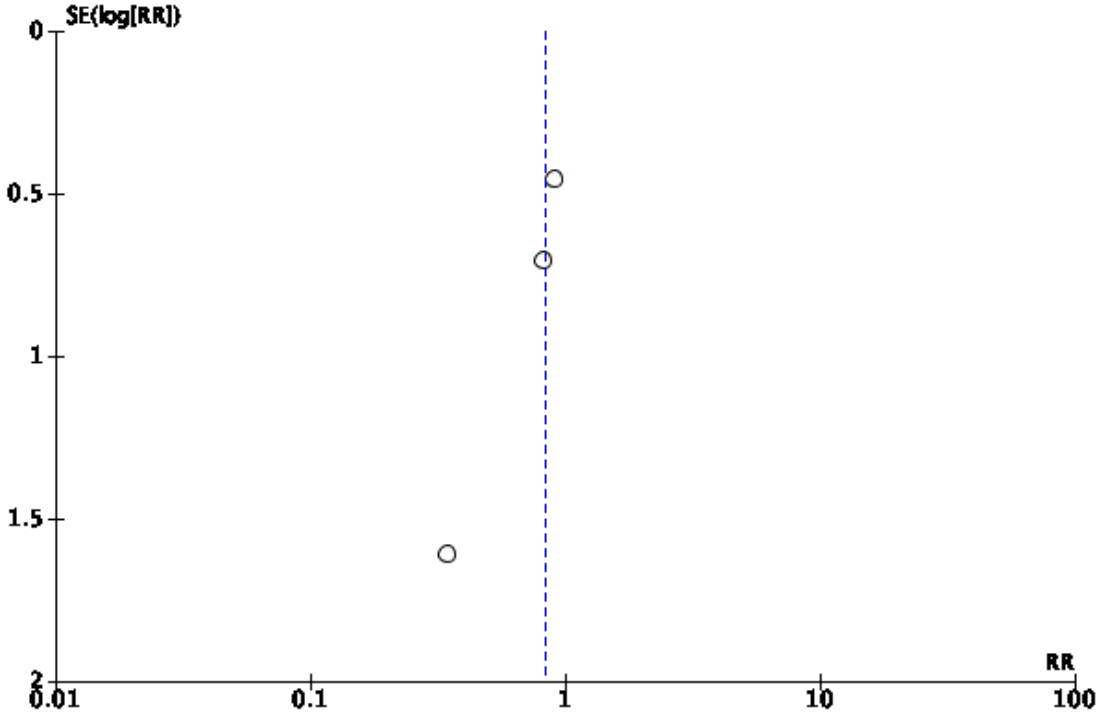

S3.3 Funnel plot of AKI for ICU-based studies

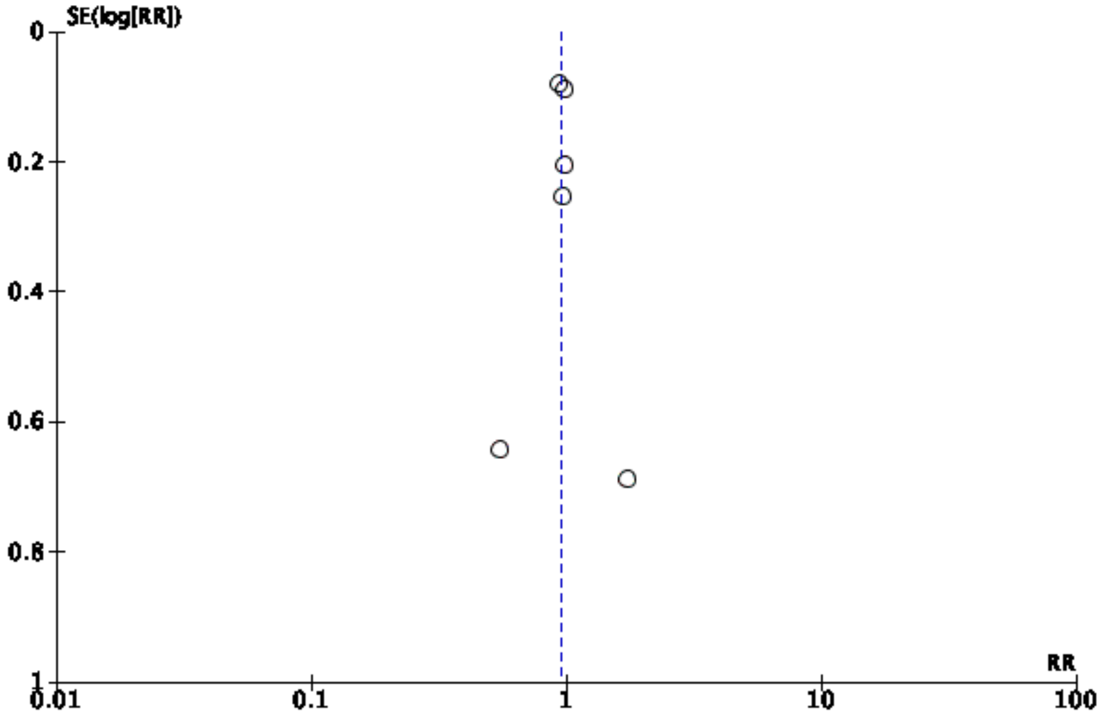

S3.4 Funnel plot of AKI for ED-based studies

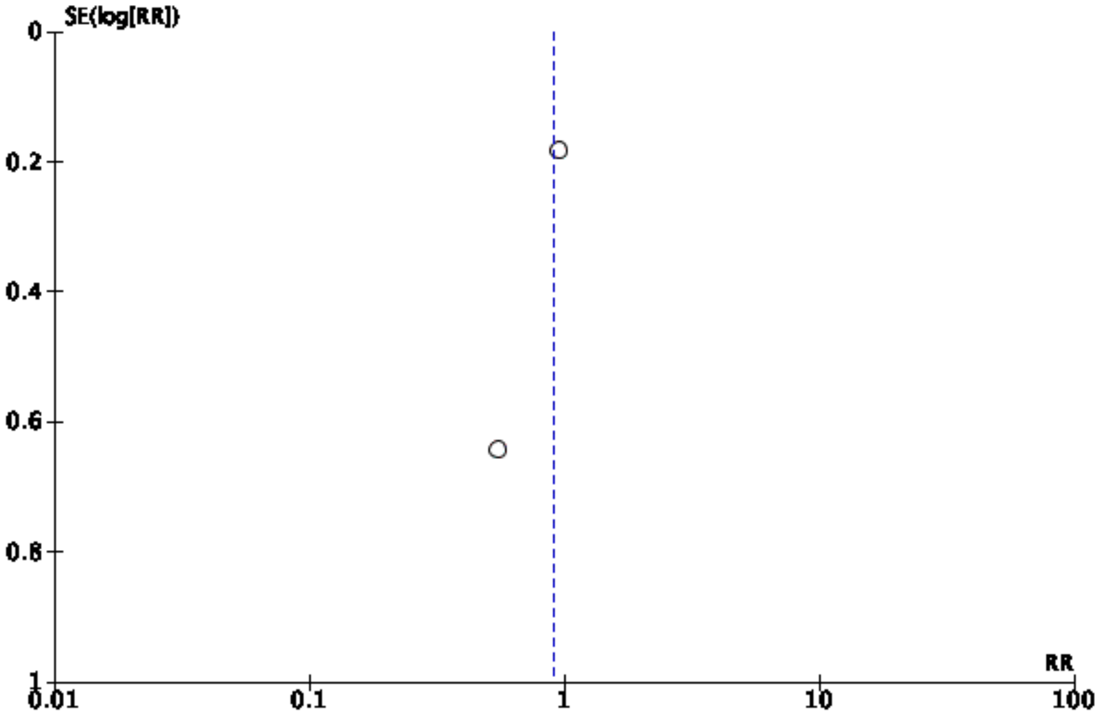

S3.5 Funnel plot of RRT for ICU-based studies

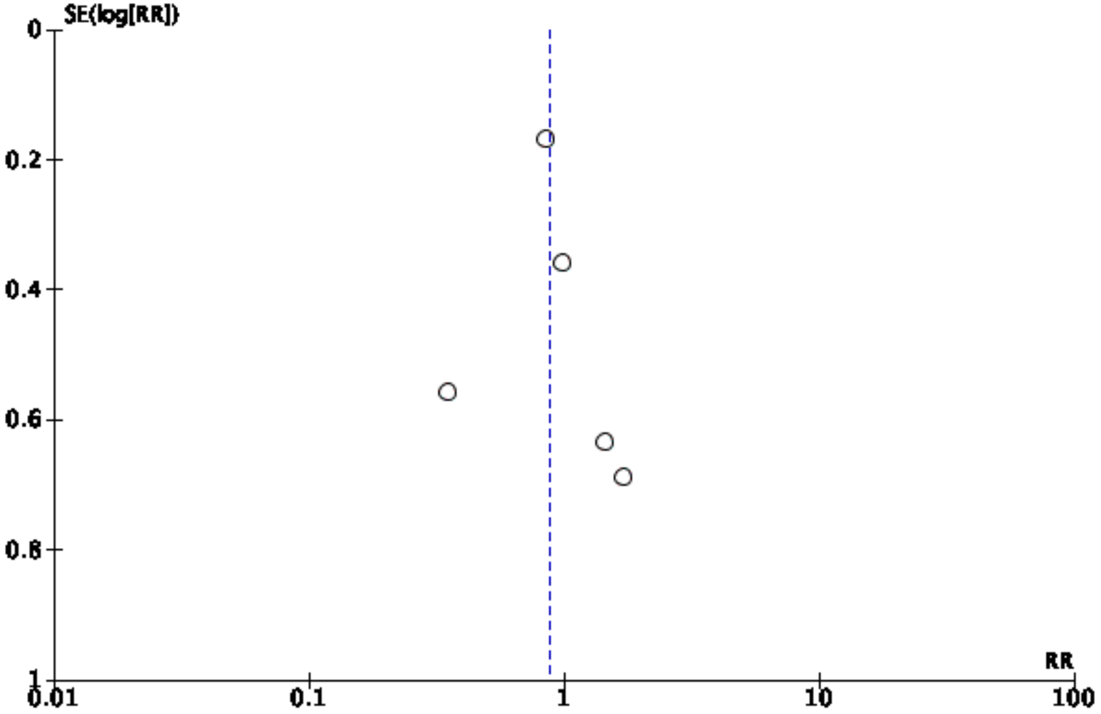

S3.6 Funnel plot of ED-based studies

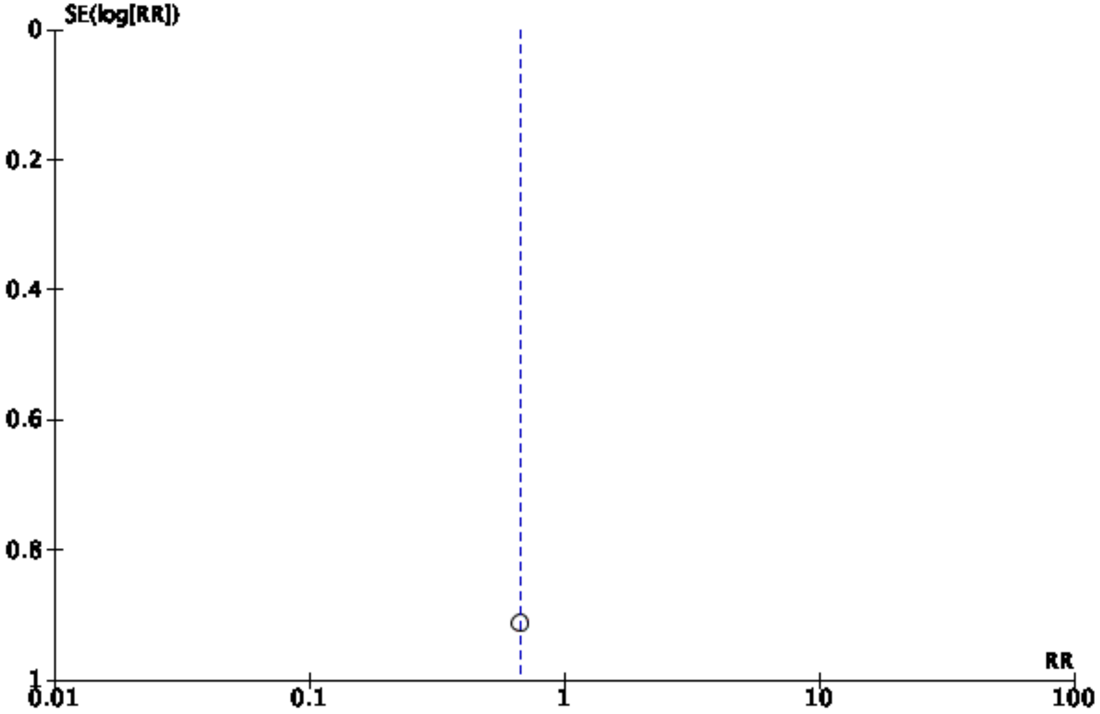

Supplement: Supplementary file 4 — Additional file 4: Figure S3. Funnel plots of included trials. [file 13054_2019_2658_MOESM4_ESM.pdf]
